# Mitochondria: It is all about energy

**DOI:** 10.3389/fphys.2023.1114231

**Published:** 2023-04-25

**Authors:** Amaloha Casanova, Anne Wevers, Santiago Navarro-Ledesma, Leo Pruimboom

**Affiliations:** ^1^ Department of Physiotherapy, University of Granada, Granada, Spain; ^2^ Faculty of Health Sciences, Melilla, Spain; ^3^ PNI Europe, The Hague, Netherlands; ^4^ Chair of Clinical Psychoneuroimmunology, University of Granada and PNI Europe, Granada, Spain

**Keywords:** mitochondria, mitochondrial dysfunction, mitochondrial metabolism, mitochondrial hormesis, hormesis

## Abstract

Mitochondria play a key role in both health and disease. Their function is not limited to energy production but serves multiple mechanisms varying from iron and calcium homeostasis to the production of hormones and neurotransmitters, such as melatonin. They enable and influence communication at all physical levels through interaction with other organelles, the nucleus, and the outside environment. The literature suggests crosstalk mechanisms between mitochondria and circadian clocks, the gut microbiota, and the immune system. They might even be the hub supporting and integrating activity across all these domains. Hence, they might be the (missing) link in both health and disease. Mitochondrial dysfunction is related to metabolic syndrome, neuronal diseases, cancer, cardiovascular and infectious diseases, and inflammatory disorders. In this regard, diseases such as cancer, Alzheimer’s, Parkinson’s, amyotrophic lateral sclerosis (ALS), chronic fatigue syndrome (CFS), and chronic pain are discussed. This review focuses on understanding the mitochondrial mechanisms of action that allow for the maintenance of mitochondrial health and the pathways toward dysregulated mechanisms. Although mitochondria have allowed us to adapt to changes over the course of evolution, in turn, evolution has shaped mitochondria. Each evolution-based intervention influences mitochondria in its own way. The use of physiological stress triggers tolerance to the stressor, achieving adaptability and resistance. This review describes strategies that could recover mitochondrial functioning in multiple diseases, providing a comprehensive, root-cause-focused, integrative approach to recovering health and treating people suffering from chronic diseases.

## 1 Introduction

### 1.1 Mitochondria targets for root cause medicine

Over the course of evolution, the fates of mitochondria and the rest of the eukaryotic cells have become intricately intertwined. The selective advantage of this endosymbiotic relationship for the host is manyfold, and additional functions are discovered rapidly, adding insight into its significance and central role in human health. As such, dysfunctions related to mitochondria can result in detrimental health consequences. Nowadays, mitochondrial dysfunction is known to be related to a broad range of diseases. From pulmonary, urinary, and metabolic pathologies to neurological and proliferative diseases (Tian et al., 2022).

Mitochondria facilitated humans to adapt and evolve by enabling flexible physiological responses to new environments (Wallace, 2015). They are an intersection point for external experiences and biological stress responses. Reciprocally, acute physiological stressors have become a vocal point for mitochondrial functioning and health in general (Navarro-Ledesma et al., 2022). Protocols, including acute physiological stressors, show promising results in the prevention and treatment of diseases, especially those strategically combining different hormetic and evolutionary interventions (Bosma-Den Boer et al., 2012; Ruiz-Núñez et al., 2013; Lane and Martin, 2015; Pruimboom et al., 2015; Pruimboom et al., 2016; Pruimboom and Muskiet, 2018). Per the low-cost nature of these interventions, they have importance at the individual and public health levels.

This study provides a theoretical framework for the expanding field of mitochondrial functions, highlighting recent insights into multiple mitochondrial disorders and their influence on the development of different pathologies. We finally describe several treatment options based on the combinations of physiological hormetic stressors on mitochondrial and, thereby, overall health (Pinna et al., 2022). Mitochondria are true integrative hubs and might be the main connector of all biological domains involved in health and handling a plethora of chronic non-communicable diseases (CNCDs).

## 2 Part I: Molecular mechanism in which mitochondria are involved

**FIGURE 1:**
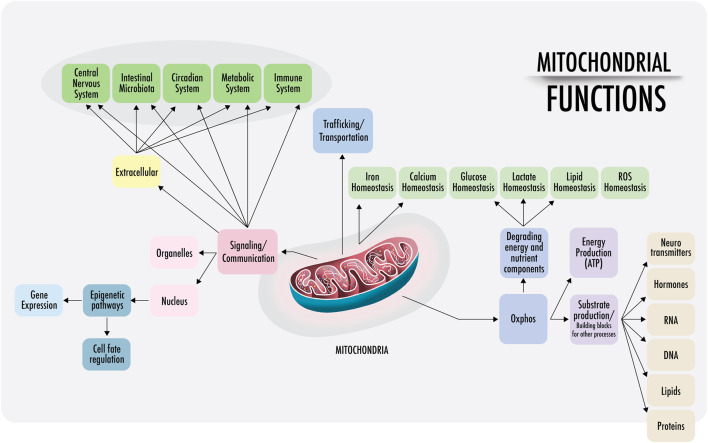
Mitochondrial functions: a visual representation of the functions of mitochondria discussed in this paper. Right: within the cell, mitochondria are anchored and transported across the cytoskeleton and cell membranes. Mitochondrial density is especially high at the perinuclear level and near the endoplasmic reticulum in most cells (also in the synaptic areas of neurons). The physical interactions of the structural domains known as mitochondria-associated membranes (MAMs) are physical contacts between organelles, such as the nucleus, lysosomes, endoplasmic reticulum, Golgi apparatus, that regulate messages through the transfer of ions and metabolites that act as a signaling center. This regulates the demands of substrate ATP and reactive oxygen species (ROS), among others. Left: intercellular communication. Cell-free mitochondria and their probable signaling functions.

As the literature states, “eukaryotes are special, and mitochondria are why” (Pinna et al., 2022). Hereafter, the various functions and characteristics of mitochondria will be discussed. The symbiosis between two prokaryotes produced the development of mitochondria and the start of all actual living organisms including plants Figure 1.

### 2.1 ATP energy production

**FIGURE 2:**
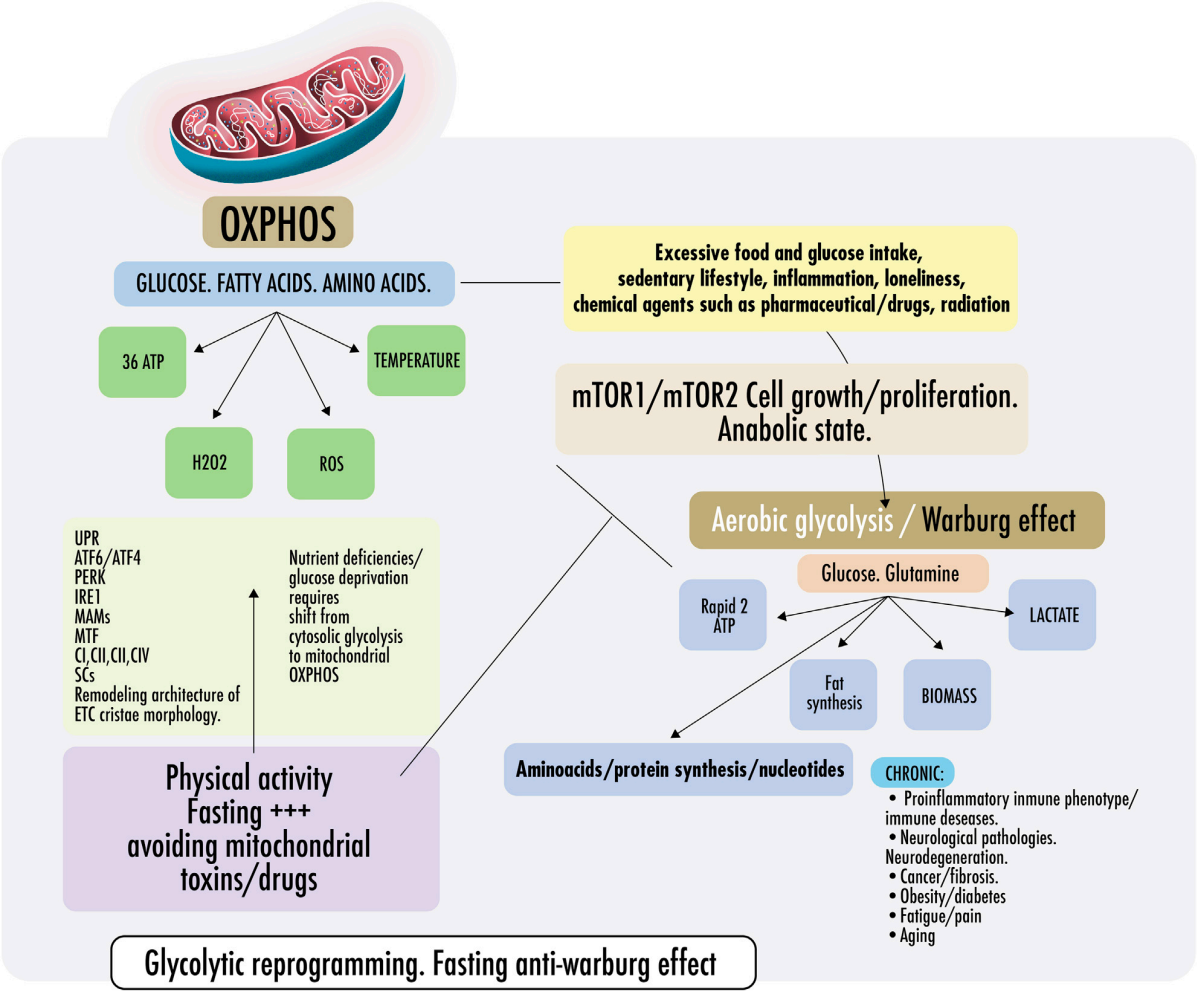
Warburg effect and anti-Warburg effect. Switch From OXPHOS to aerobic glycolysis. Warburg effect and anti-Warburg effect. Under physiological conditions, cells can change from mitochondrial respiration to cytosolic respiration. Mitochondria allow the efficient production of ATP and regulate temperature, producing ROS (OXPHOS). In the presence of nutrients, cytosolic glycolysis permits cell repair/proliferation without oxidation, which facilitates the production of amino acids, fats, and nucleotides, among others. It is important to keep these processes alternate and intermittent. Right: in pathological states, some risk factors are mentioned, such as inflammation, loneliness, glucose surplus, and some medications, which induce a metabolic change from OXPHOS to aerobic glycolysis directed by the mechanistic target of rapamycin if persistently maintained (mTORC1/mTORC2). Both continously activated result in failure of the lysosomal and autophagy mechanisms inducing persistent anabolic state, fibrosis, excess proliferation, and changes in the immunometabolic phenotype that could result in CNCDs. Left: to restore physiological states and recover OXPHOS, exercise and fasting achieve an anti-Warburg effect. During nutrient deprivation, cells demand OXPHOS to increase bioenergetic capacity by driving a decrease in fission and remodeling in the electron transport chain (ETC) or cristae morphology. It is not yet clear, but probably, ETC architecture remodeling is driven because of endoplasmic reticulum ER stress during fasting because of the disruption of protein folding and glycosylation. An imbalance in protein folding capacity starts the unfolded protein response (UPR) mechanism, activating transcription factor (ATF6/ATF4), protein kinase R- (PKR-) like ER kinase (PERK), and inositol-requiring enzyme (IRE1) to re-establish ER homeostasis and maintain protein folding. Physical contact sites termed mitochondria-ER-associated membranes (MAMs) regulate calcium homeostasis, mitochondrial fission, lipid metabolism, autophagy, and inflammasome activity. ER communicates with the OXPHOS system to increase ATP supply and promote protein homeostasis. Exercise also induced mitochondrial cristae remodeling or shaping, improving the activity of respiratory chain complexes (CI, CII, CIII, and CIV) in the inner membrane and mitochondrial respiratory efficiency. Exercise impacts the stoichiometry of the SCs, enhancing the efficiency of electron flux by segmentation of the CoQ, improving the stability of the individual respiratory complexes, and avoiding ROS excess.

Figure 2 Cells require a constant supply of energy to generate and maintain the biological organization that keeps them alive and functioning. Adenosine triphosphate (ATP) is the source of energy for most cellular processes (Pinna et al., 2022). Mitochondria are the main energy production sites, converting substrates into ATP; the breakdown of nutrients into energy is called mitochondrial oxidative phosphorylation (OXPHOS) (Herzig and Shaw, 2018). Without mitochondria, humans would be dependent on the relatively energy-inefficient process of aerobic glycolysis (discussed below), a cytosolic process resulting in two ATPs per molecule of glucose. In aerobic glycolysis, glucose is converted into lactate through the reaction with nicotinamide adenine dinucleotide (NAD+). This reaction forms cytosolic reduced nicotinamide adenine dinucleotide (NADH) and lactate by lactate dehydrogenase (LDH) with a continuous reversal from lactate to pyruvate (Glancy et al., 2021). In contrast, mitochondrial OXPHOS activity yields an energy production that exceeds 30 molecules of ATP per molecule of glucose. As the body cannot easily store ATP, mitochondrial OXPHOS activity is essential for health and, therefore, should dominate cell metabolism most of the time (Bonora et al., 2012)**.**

Several important processes in metabolism are found within the mitochondrial matrix, including pyruvate decarboxylation by the pyruvate dehydrogenase complex (PDC), which converts pyruvate to acetyl coenzyme A (acetyl-CoA), and the same holds for fatty acid beta-oxidation enzymes and the production of acetyl-CoA from free fatty acids. All provide reducing equivalents to the tricarboxylic acid (TCA) cycle and OXPHOS (Herzig and Shaw, 2018).

The respiratory chain activity sequentially transfers electrons between four major multi-enzymatic complexes dispersed in the inner mitochondrial membrane (IMM) (Enriquez and Lenaz, 2014). Of recent interest are mitochondrial supercomplexes (SCs), evolutionarily conserved structures that are products of the association of mitochondrial respiratory chain (MRC) multi-heterodimeric complexes (I–IV). These structures are thought to provide functional advantages in the electron transfer process. SCs differ among species and tissues, depending on the metabolic and physiological conditions, as well as on the lipid content of the IMM. The most abundant SC in mammals is I + III_2_ + IV_1_, known as the respirasome and organized through phospholipid activity or the availability in the IMM. Stable SCs are essential for mitochondrial functioning, and phospholipids, such as cardiolipin and probably phosphatidylethanolamine, prevent the destabilization of SCs and possible mitochondrial dysfunctions (Lobo-Jarne and Ugalde, 2017; Nesci et al., 1103).

Other important participants in the respiratory chain are two mobile components acting as co-substrates: the lipophilic ubiquinone, coenzyme Q (CoQ), embedded in the membrane lipid bilayer, and the hydrophilic heme protein cytochrome c (cytc) located on the external surface of the IMM (Lobo-Jarne and Ugalde, 2017; Nesci et al., 1103).

Different theories exist on the organization of the respiratory chain and its components. Opposed to other models, the plasticity model describes the respiratory chain as a dynamic organization (Enriquez and Lenaz, 2014). Individual complexes and SCs are thought to participate in the electron transfer collectively and individually. Altogether, the knowledge about the way the respiratory chain functions is constantly increasing, and the same holds for the mechanisms of their dysfunction and their role in the development of (chronic) diseases and aging. Nevertheless, the exact structural organization of its components remains to be fully elucidated (Lobo-Jarne and Ugalde, 2017; Nesci et al., 1103).

### 2.2 Lactate shuttling

Recently, the role of lactate as an energy source has become more apparent, as well as the function of mitochondria in its metabolism. Lactate was considered a metabolic waste product with unfavorable effects. However, this view has changed, with mitochondrial L-lactate dehydrogenase (m-L-LDH) being of vital importance for the metabolism of many organs. It oxidizes lactate into pyruvate, enabling it to become a substrate for mitochondrial respiration and, thus, energy supply (Young et al., 2020).

Besides enabling lactate to become a metabolic substrate, mitochondria have a significant role in lactate shuttling. Lactate shuttling enables the exchange of lactate between producer (driver) cells and so-called consumer (recipient) cells (Brooks et al., 2022). The lactate shuttle is propelled from white fibers (drivers) to red fibers (lactate users) thanks to their high mitochondrial density (Brooks, 2002). An essential shuttle exists between muscle lactate produced during movement/exercise (driver) and its transfer to the heart and brain (receivers) that use lactate as an important energy source. Lactate from muscular cells can also be used in the liver, kidneys, lungs, sperm, and many other organs, making lactate an essential energy source, opposite to the common opinion that lactate is a toxic metabolite (Brooks, 2018; Brooks, 2020a; Brooks, 2020b; Brooks et al., 2022). Furthermore, the exchange between astrocytes and neurons is common, where lactate is metabolized and excreted by astrocytes and subsequently actively consumed and oxidized by neurons. In addition to intercellular shuttling, intracellular shuttling is possible. This refers to the exchange of lactate between cell constituents, such as the cytosol and the mitochondria or peroxisomes. Mitochondria are of essence here, as the shuttling depends on pH and concentration gradients. These are created by mitochondria in recipient (user) cells (Brooks, 2018; Brooks, 2020a; Brooks, 2020b; Brooks et al., 2022). Mitochondria contain a collection of proteins bound to the IMM and organic molecules, which electrons pass through in a series of redox reactions. The oxidation of NADH and FADH2 causes the release of energy, and the energy released forms a pH gradient of protons. Lactate transport is pH-sensitive and proton-dependent and subsequently occurs through facilitated exchange along the pH and concentration gradients, involving a family of proton-driven lactate transport proteins called monocarboxylate transporters (MCTs) (Payen et al., 2020).

Disorders in lactate metabolism are related to multiple diseases, of which perhaps cancer is the most evidenced. Lactate produced by aerobic glycolysis in cells suffering from chronic hypoxia is a so-called oncometabolite implicated in tumor epigenetic regulatory activity, tumor angiogenesis, and lipid metabolisms favoring anabolic and catabolic activity in the tumor (de la Cruz-López et al., 2019; Kes et al., 2020; Ippolito et al., 2022).

Cancer is conventionally considered a disease caused by genetic mutations, although some authors put this hypothesis in serious doubt, suggesting that in-depth knowledge of cancer metabolism is essential for the development of successful cancer therapies (Duraj et al., 2021; Seyfried and Chinopoulos, 2021; Sainero-Alcolado et al., 2022). Perhaps the most convincing evidence of cancer being a metabolic disease comes from studies conducted with cancer cells, in which the implantation of healthy mitochondria suppresses the further development of cancer cells, although the cell nucleus shows an abundant number of mutated oncogenes. Other studies showed that cancer develops when “cancerous” mitochondria are implanted in a healthy cell without any genetic mutations (Gyamfi et al., 2022). Many oncometabolites causing mitochondrial disorders and possibly initiating the cancer process have been identified, and as aforementioned, lactate is one of the most ubiquitous oncometabolites in cancer (Kes et al., 2020; Shegay et al., 2022). Aerobic glycolysis in tumor cells is important, but dynamic adjustment of mitochondrial RNA modification directly contributes to tumor malignancy and metastasis, shaping tumor metabolic plasticity (Delaunay et al., 2022). We will discuss the role of mitochondria and cancer in more depth in part II.

### 2.3 Maintenance of homeostasis

Mitochondria are highly mobile *in vivo* in neurons and *in vitro* in multiple cell lines (Green et al., 2022). The maintenance of mitochondrial motility is believed to be vital to cellular function. Trafficking ensures ATP supply at local sides of demand and calcium buffering. They are involved in cell differentiation and cell division to ensure proper inheritance (Debattisti et al., 2017), the efficiency of embryological development, and neurodevelopmental and immunological efficiency (Walker and Moraes, 2022). Mitochondria and their viability are primarily dependent on the level of production of cytosolic reactive oxygen species (ROS), the calcium balance, and adenosine monophosphate protein kinase (AMPK) signaling (Walker and Moraes, 2022). Within the cell, mitochondria are anchored and transported across the cytoskeleton and cell membranes. They use several anchoring proteins; for example, syntaphilin is an anchoring protein found in nerve cells and necessary for maintaining mitochondrial density and viability (Kraft and Lackner, 2018). Mitochondrial density is especially high at the perinuclear level and close to the endoplasmic reticulum (ER) in most cells. In neurons, mitochondrial density is high in synaptic areas to guarantee energy supply and efficiency of nerve transmission (Walker and Moraes, 2022). Chronic ROS stress on mitochondria can cause aging, affecting neuronal survival. Again, syntaphilin protects against malfunctioning by eliminating damaged mitochondria, and adequate removal of those mitochondria prevents axonal deterioration (Lin et al., 2017).

Homeostasis depends on the availability of sufficient energy (ATP). Mobile mitochondria can pause in regions that have a high metabolic demand. Mitochondrial distribution is often quite heterogeneous, showing enrichment at sites with high energy demand (Cunniff et al., 2016). Muscles are important regions whose ATP/ADP ratio affects contraction capacity, whereas ADP slowly dissociates from the motor to act as an inhibitor of motility (Debattisti et al., 2017). Thereby, active infiltration of mitochondria leads to the development of edge and protrusive cell structures supporting localized ATP production (Cunniff et al., 2016). ROS, either derived from an extracellular source or intracellularly generated, controls a dose-dependent mitochondrial distribution and function through the p38α pathway (Brooks et al., 2022). P38α specifically and reversibly decreases mitochondrial motility, and the role of atypical activation of p38α is part of the pathophysiology of retinal diseases, cardiovascular diseases, neurodegenerative diseases, diabetes, and cancer (Burton et al., 2021).

The fact that mitochondrial motility is induced by physiologically relevant doses of H_2_O_2_ and is further rapidly reversed by the removal of H_2_O_2_ supports the idea that ROS might work as a physiological regulator of mitochondrial distribution, temporally decelerating the migration of these organelles when and where it is required. In this context, H_2_O_2_ becomes a signaling molecule involving mitochondrial and cell motility through the dynamic rearrangement of actin networks. Therefore, it seems logical that H_2_O_2_ levels are increased in migrating cells compared to stationary ones (Pak et al., 2020). This process may enable the recruitment of additional mitochondria at the site of elevated ROS production and where they serve as scavengers and propagators and possibly protect against the development of pathophysiological conditions (Debattisti et al., 2017).

#### 2.3.1 Calcium and its availability show a high impact on mitochondrial functioning

Diminished mitochondrial motility in the region of the Ca^2+^ rise promotes the recruitment of mitochondria to enhance local Ca^2+^ buffering (Yi et al., 2004). Both deficient as excessive Ca^2+^ uptake is related to different chronic disorders. Excessive mitochondrial Ca^2+^ uptake has been shown to induce cell death in patients with Alzheimer’s disease (AD) (Calvo-Rodriguez et al., 2020), whereas deficient Ca^2+^ uptake plays an important role in the pathophysiology of Parkinson’s and possibly amyotrophic lateral sclerosis (ALS) (Calvo-Rodriguez et al., 2020).

Mitochondria participate in intracellular Ca^2+^ signaling as modulators, buffers, and sensors (Calvo-Rodriguez et al., 2020). They can store and release Ca^2+^ and thus influence the shape, frequency, and amplitude of Ca^2+^ spikes in the cytosol (Wacquier et al., 2016).

Calcium handling by mitochondria is a key feature in cell life. The cell cycle control machinery must ensure perfect genome duplication and cell division, the basis for self-replication. Calcium-based signaling is a universal mechanism through which extracellular messengers modify the activity of target cells. Cells can decode Ca^2+^ signals based on the characteristics of intracellular changes in Ca^2+^ concentration (amplitude, duration, frequency, and location). Four fundamental, intimately related, and interdependent processes are responsible for cell life: survival, proliferation, differentiation, and death (Danese et al., 2021). Ca^2+^ is essential in each of these processes, especially the impact of Ca^2+^ homeostasis on cell death mechanisms (Contreras et al., 2010; Danese et al., 2021; Garbincius and Elrod, 2022). Mitochondrial Ca^2+^ uptake primarily depends on the mitochondrial Ca^2+^ uniporter (MCU). MCU-mediated effects drive cell cycle, ATP, and ROS production (Zhao and Pan, 2021). As aforementioned, disturbances of Ca^2+^ buffering can lead to many neurodegenerative disorders. Therefore, the regulation of Ca^2+^ by mitochondria should be an important target for the primary and secondary prevention of a wide range of neurodegenerative diseases (Rodríguez et al., 2022).

Trafficking also contributes to the cleansing and replenishment of mitochondria in the periphery (Schwarz, 2013). Postmitotic cells need to survive for the lifetime of an organism (Schwarz, 2013). Studies show that the lifetime of mitochondrial proteins varies from weeks to several years (Krishna et al., 2020). As this usually does not cover the lifetime of a cell, replenishment is necessary. Thus, constant, efficient turnover of mitochondria is important to maintain health during the entire cell life cycle. This includes the clearance of older, damaged components and the delivery of new materials (Schwarz, 2013).

A recent discovery is the existence of cell-free mitochondria. This discovery could have significant physiological consequences that need to be elucidated. Intact cell-free mitochondria oversee normal energy production and cellular metabolism. The free mitochondria may act as signals in cell-to-cell communication. An estimated 200,000–3,700,000 functional mitochondria were found per ml of extracted plasma. These are a new class of signaling organelles involved in complex regulatory activities and intercellular communication (Al Amir Dache et al., 2020). Whether functional blood-borne mitochondria serve as sentinels to maintain homeostatic metabolic activities and as a reserve for essential cellular functions remains to be confirmed. It has been suggested that these cell-free mitochondria present immunologically active proteins, such as programmed cell death-ligand 1 (PD-L1) and CD270, both associated with the upregulation of CD4 + T cells, CD8 + T cells, and reduced concentrations of pro-inflammatory cytokines (Stefano and Kream, 2022).

### 2.4 Building blocks and storehouses

Besides providing energy and the implications of their ability to move, mitochondria contribute to the production of multiple macromolecules, such as lipids, proteins, DNA, and RNA (Spinelli and Haigis, 2018). Mitochondria are an important site for the synthesis of steroid hormones and key neurotransmitters (Pak et al., 2020; Burton et al., 2021). Acetylcholine is an example of mitochondrial-produced neurotransmitters, the primary neurotransmitter of the autonomic system and the chief neurotransmitter of the parasympathetic nervous system (Skok, 2022). A second example is glutamate, a critical excitatory neurotransmitter in the central nervous system (CNS) and essential for multiple brain functions (Scalise et al., 2017a). Thereby, mitochondria play a critical role in the *de novo* synthesis of other key neurotransmitters, such as noradrenaline (NA), gamma-aminobutyric acid (GABA), and serotonin (Guo et al., 2017).

Even melatonin is produced in neuronal mitochondria (Carter et al., 2021). An interesting study, including 12 subjects, investigated the effects of lithium (Li), valproate (VPA), and lamotrigine (LTG) on humans (Danese et al., 2021). These are established therapies for bipolar disorder (BD) and other mood disturbances; however, their mechanisms of action have not yet unraveled. The results showed that the treatment with Li, VPA, and LTG led to enriched transcriptional signatures favoring the OXPHOS pathways. In addition to shared genes, they found that Li exposure was associated with 554 genes enriched for OXPHOS pathways and thermogenesis (Osete et al., 2021).

It is important to state that neurotransmitter and hormone production can only occur when mitochondria use OXPHOS as a metabolic pathway; mitochondrial dysfunction and loss of OXPHOS capacity can cause multiple hormonal and neurotransmitter dysfunctions and diseases (Suliman and Piantadosi, 2016; Danese et al., 2021).

Finally, mitochondria import iron and, in turn, store, traffic, and supply the cell with heme. Heme is necessary to assemble cytosolic and nuclear proteins that contain iron–sulfur groups (Dietz et al., 2021). Iron–sulfur groups are precursors for hemoglobin, necessary to bind oxygen in the bloodstream. Therefore, mitochondria have a significant role in iron homeostasis, strictly regulating cellular iron levels, heme, and intracellular ferritin level (Ward and Cloonan, 2019).

### 2.5 Mitochondria and biological rhythms

One of the most underestimated risk factors for the development of chronic diseases in the patient population is a disturbance of the circadian rhythm. Circadian misalignment is associated with numerous diseases, including neurodegenerative diseases (Sardon Puig et al., 2018; Abbott et al., 2020), cardiovascular disorders, and even cancer (Sardon Puig et al., 2018; Logan and McClung, 2019; Abbott et al., 2020). Sleep quantity and quality are influenced by multiple environmental factors, such as artificial light, meal timing, and socialization (Abbott et al., 2020). Mitochondrial functioning is much affected by the circadian rhythm, circadian misalignments affect mitochondrial viability, and mitochondrial dysfunction affects the circadian rhythm (Sardon Puig et al., 2018). The circadian timing system is hierarchically structured, with the suprachiasmatic nucleus (SCN) as a master pacemaker of all organs, including the brain. The SCN synchronizes subsidiary oscillators—clock proteins coded by clock genes—in almost every tissue and cell of the body. The oscillating chemical signals released by the SCN influence intracellular clock proteins and modulate almost all physiological processes in mammals (Garbincius and Elrod, 2022). The central mechanism responsible for the accurate function of clock genes is based on a transcription/translation negative and positive feedback loop (Acuña-Castroviejo et al., 2017a; Ashton et al., 2022).

The positive branch of rhythmic genes consists of circadian locomotor output cycles kaput (CLOCK) and brain and muscle ARNT-like/basic helix–loop–helix ARNT-like 1 (BMAL1). Other examples are neuronal PAS domain protein 2 (NPAS2) and aryl hydrocarbon receptor-like nuclear translocator (ARNTL)/BMAL1 or aryl hydrocarbon receptor-like nuclear translocator (ARNTL2)/basic helix–loop–helix-like RNAT 2 (BMAL2). Negative regulators include period (Per1, Per2, and Per3) and cryptochrome (Cry1 and Cry2). The PER and CRY proteins heterodimerize and repress their own transcription by interacting in a feedback loop with CLOCK/ARNTL complexes. In summary, PER1/2/3 and CRY1/2 are transcriptional repressors and interact with the CLOCK, NPAS2-ARNTL/BMAL1, or ARNTL2/BMAL2 heterodimer, inhibiting their activity (Acuña-Castroviejo et al., 2017a; Carmona et al., 2020; Thakur et al., 2020; Gabriel et al., 2021).

Most mechanisms maintaining an optimal circadian rhythm are related to normal mitochondrial functioning in the SNC (Aguilar-López et al., 2020), whereas the opposite is true, meaning that mitochondrial disorders affect sleep and circadian rhythm (Scrima et al., 2016; Sardon Puig et al., 2018). Mitochondria can regulate energy, subsequently allowing the regulation of the central and peripheral clocks.

Mitochondria produce melatonin in neuronal mitochondria only when energy is delivered by OXPHOS (Carter et al., 2021). The main function of melatonin is to provide a time cue to the SCN (Stefano and Kream, 2022). Furthermore, mitochondria produce NAD+, which is necessary to activate SIRT1 and SIRT3. Sirtuins have been reported to regulate central and peripheral clocks. Sirtuins further modulate the circadian epigenome and maintain specificity in transcriptional control (Stefano and Kream, 2022). Indeed, pharmacological inhibition of the mitochondrial OXPHOS system resulted in dramatic deregulation of the rhythmic clock-gene expression. A similar result was attained with mitochondrial DNA (mtDNA) depleted cells (Scrima et al., 2016).

Mitochondrial dynamics and changes in mitochondrial architecture are reported to influence circadian rhythmicity (Sardon Puig et al., 2018). Crosstalk between circadian clocks and mitochondria is given by the fact that circadian clocks regulate the biosynthesis of NAD+ and thus the mitochondrial capacity for energy production (Aguilar-López et al., 2020).

On a molecular level, there are several synchronization pathways between mitochondria and circadian molecular controllers. The clock protein CRY (Sirt1 and 3 dependent) influences mitochondrial activity, reduces gluconeogenesis, and favors substrates for OXPHOS through glycolysis, enhancing ATP production (Aguilar-López et al., 2020). AMPK detects the increase in energy and reduces the activity of CRY and the production of ATP by the mitochondria. Inactivation of CRY releases BMAL/CLOCK, which initiates a new cycle enhancing CRY and RORα expression, whereas the latter, with SIRT1/PGC1α, further increases BMAL1 expression and mitochondrial biogenesis. PGC-1α, the main regulator of mitogenesis, constitutes a link between clock genes and metabolism. SIRT1 and PGC-1α in the mitochondria regulate energy metabolism and activate mitochondrial transcription factor A (TFAM). This, in turn, regulates mtDNA copy number and transcriptional activity (Acuña-Castroviejo et al., 2017a; Poole and Ray, 2022). All resumed data show the important crosstalk between circadian rhythm and mitochondrial function, which is essential for understanding health and disease.

### 2.6 Communication: Mitochondria as an integrative hub

Evolutionary pressure led to multiple communication pathways between mitochondria and other organelles of the host cell and other cells. Most of those pathways are critical for cell homeostasis and are recognized as cellular and organismal signaling hubs (Shen et al., 2022a). The exemplary role of Ca^2+^ signaling and its interaction with circadian rhythms have been discussed earlier in this review.

Intracellular communication depends on circadian rhythm, metabolism, intestinal microbiota, and the immune system, but they are subordinate to mitochondrial functioning and communication. Mitochondria seem to regulate the back and forward trafficking of information from one domain to the other. As such, mitochondria might be a hub within the circadian clock-metabolism-intestinal-microbiota-immune system network (Aguilar-López et al., 2020). If these data are confirmed *in vitro* and *in vivo*, targeting mitochondria with evidenced-based interventions could be primary for the treatment of multiple chronic diseases related to this network. Those interventions could include exercise, regulation of gut microbiota, and cognitive training, all connected through mitochondrial functions (Clark and Mach, 2017; Zhu et al., 2022a).

Mitochondria are increasingly recognized as information hubs that sense cellular changes and transmit messages to other cellular components. They dynamically sense the constantly changing intra- and extracellular environmental milieu and relay messages to other subcellular compartments. One way of communication is facilitated by physical interactions of structural domains known as mitochondria-associated membranes (MAMs). These MAMS are physical contacts between organelles, such as nucleus, lysosomes, ER, and Golgi apparatus (Benayoun and Lee, 2019a; Wang et al., 2021a; Lv et al., 2022). They regulate messages by transferring ions and metabolites, acting as a signaling hub, and controlling many cellular functions. Alterations of these MAMS are associated with several pathologies (López-Crisosto et al., 2015; Annunziata et al., 2018; Eysert et al., 2020).

#### 2.6.1 Mito-nuclear communication

At the moment in evolution when mitochondria became cellular endosymbionts, they transferred most of their genomes to the nucleus of the host cell (eukaryote), thereby massively reducing their number of genes (Walker and Moraes, 2022). Mitochondrial genes are encoded in the cell nucleus to produce the proteins of the outer and inner membrane, those of the intermembrane space, and most of the proteins of the mitochondrial matrix. These nuclear genes encode the proteins to be synthesized in the ribosomes. The precursor proteins are recognized by mitochondrial receptors, imported by multiple complex systems, assembled and translocated to integrate the internal–external membranes, or released into the matrix (Millichap et al., 2021).

Nuclear respiratory factors 1 and 2 (NRF1/NRF2) are transcription factors encoded in the nucleus signaling more than 250 genes. Many of them are mitochondrial genes. NRF1 regulates gene expression related to mitochondrial respiration, heme formation, and the import and assembly of mitochondrial proteins. NRF2 influences the transcription of genes related to the electron transport chain (ETC) activating complexes II (succinate dehydrogenase) and IV (cytochrome oxidase) (Millichap et al., 2021; Walker and Moraes, 2022).

A relevant function of NRF1/NRF2 is that they activate mitochondrial transcription factor A (TFAM) and peroxisome proliferator-activated receptor gamma coactivator 1α (PGC-1α). These are master regulators responsible for mitochondrial biogenesis and mitochondrial DNA stability (Benayoun and Lee, 2019a; Muri and Kopf, 2020; Calabrese and Kozumbo, 2021). NRF efficiency is necessary to regulate ROS signaling, and ROS is involved in the initiation and progression of any disease (Biagiotti et al., 2019; Bhandari et al., 2021a; Morris et al., 2021; Ulasov et al., 2021; Vashi and Patel, 2021).

Mitochondrial signals that activate nuclear responses are called “retrograde signals.” Their function is to improve energy efficiency, monitor the cell cycle, respond to cellular stress, and eliminate dysfunctional organelles, among others (Yang and Kim, 2019).

A variety of stimuli and pathways fall under the retrograde signaling umbrella. An example of the importance of mtDNA is given when depletion leads to calcium dysregulation and susceptibility to a range of diseases. mtDNA depletion syndromes are normally caused by mutations in mitochondrial genes and are very deleterious for patients suffering from mtDNA syndromes (El-Hattab and Scaglia, 2013). Calcium dysregulation can be responsible for the release of pro-inflammatory factors, cell proliferation factors, and anti-apoptotic factors (Patergnani et al., 2020). A subsequent increase in ROS, caused by mitochondrial damage or hypoxia, will induce a further hypoxic nuclear response to the increased respiration and mitochondrial and nuclear enzymatic antioxidant expression (Hernansanz-Agustín and Enríquez, 2021).

Mitochondrial products, such as acetyl CoA, α-ketoglutarate, succinate, fumarate, and FAD/FADH, will induce specific and targeted chromatin modifications and change transcriptional silencing or gene expression. NADH/NAD+, as a residue of metabolic activity, induces nuclear transcription for mitogenesis, fatty acid oxidation, DNA repair, and DNA modifications (Gyamfi et al., 2022).

Many cellular functions can be altered by mitochondrial signals. Activation of transcription factors by mitochondria leads to the translocation of transcription factors to the nucleus (Soledad et al., 2019; Walker and Moraes, 2022).

Mitochondrial-derived peptides (MDPs) are a newly recognized form of retrograde signaling substances contributing to the adaptive stress response; one of the MDPs, mitochondrial 12S ribosomal RNA type c open reading frame (MOTS-c) is a peptide with metabolic functions encoded within the mitochondrial 12S ribosomal RNA gene. MOTS-c reacts to ROS and low glucose by signaling nuclear genes to regulate cell balance (Benayoun and Lee, 2019b). Other important functions of MDPs are related to the acute stress response, for instance, caused by mitochondrial perturbation during exercise, showing protective functions and an increase in insulin sensitivity. Eight MDPs have been identified, and the use of three of them (humanin, MOTS-c, and SHLP2) (Merry et al., 2020) in rodents showed surprising positive effects related to insulin sensitivity and protection against aging processes (Merry et al., 2020). Murine animal models suggest that a decrease in MOTS is related to metabolic diseases. However, human studies show that a decrease in MOTS is differentially regulated in the skeletal muscle and plasma of healthy males during aging, suggesting that the changes in MOTS-c levels result from normal aging and do not indicate disease development (D'Souza et al., 2020). Further research on humans is necessary to show the relevance of MDPs in diseases such as type 2 diabetes and other metabolic disorders.

Mitochondrial-derived non-coding RNAs (ncRNAs) also fall under the retrograde signaling umbrella (Zhang et al., 2019). ncRNAs are a group of ribonucleic acids that are ubiquitous in the body and do not encode proteins. They have emerged as important regulatory factors in almost all biological processes. ncRNAs play a critical role in the epigenetic regulation of gene expression at the transcriptional and post-transcriptional levels. Recent evidence indicates that ncRNAs are messengers between the nucleus and the mitochondria (Vendramin et al., 2017). Common regulatory mechanisms of ncRNAs are the modulation of target genes, silencing mRNA translation or inhibition of mRNA transcription by targeting transposons, and inhibition of transcription by targeting specific loci. ncRNAs are modulators of the mitochondrial proteome. Mitochondria-localized miRNAs (mitomiRs) directly regulate mitochondrial gene expression. ncRNAs also hide small open reading frames (sORFS), encoding for small functional peptides called micro peptides (Gusic and Prokisch, 2020). Mitochondrial dysfunction leads to several diseases, such as cardiovascular diseases, cancers, and neurodegeneration. The cell/tissue-specific expression of mt-ncRNAs suggests that they could be important in mitochondria-related diseases. More in-depth knowledge about the ncRNA regulatory network would contribute to a better understanding of the etiology of mitochondrial-related diseases and lead to novel diagnostic and therapeutic approaches (Liu and Shan, 2021)

It seems clear that the complete transcriptome of mitochondria plays an essential role in the maintenance of mitochondrial viability and healthy aging. Further research is needed to see if interventions with the transcriptome as a target can help patients suffering from a wide range of chronic diseases related to mitochondrial dysfunction.

## 3 Part II: Mitochondrial dysfunction and its involvement in disease—Main dysregulated pathways in chronic diseases

### 3.1 Genetic alterations and mutations

Our review focuses on acquired dysfunctions of mitochondria and their influence on health and disease. Congenital mutations and their role in the development of chronic diseases fall out of the scope of this manuscript. Nevertheless, we will highlight some mutational changes to complete the knowledge for the reader. Mitochondrial dysfunctions are part of a group of multisystemic disorders with possible genetic alterations. Certain pathologies are linked to mutations in mtDNA, whereas others result from mutations in nuclear genes.

Mitochondrial DNA differs from nuclear DNA. Noteworthy is its maternal inheritance with a multicopy nature. There are thousands to hundreds of thousands of copies within each cell (polyplasmy). In a healthy situation, copies of mtDNA are identical (homoplasmy). Wild-type (wt) mtDNA and mutant mtDNA species frequently cohabitate in human cells, referred to as heteroplasmy. This phenomenon is commonly linked with pathological outcomes. Mutations of mtDNA can have an effect of 0%–100%, with an estimated mutation rate of 7–10 times higher than nuclear DNA. The phenotypic expression and the consequences of the mutations depend on the remaining amount of wt-mtDNA, the functionality of the mitochondria, the location in the cell, and their capacity to produce ATP in relation to the demand of the tissue. In humans, drastic changes can appear at the level of heteroplasmy in the next generation. These could either be mitochondrially transmitted adaptations or mtDNA copy numbers that flow from one generation to the following via the female germ line (Rai et al., 2018; Maude et al., 2019; Zeviani and Viscomi, 2022).

Heteroplasmy has significant implications for aging, can cause conflicts in metabolic regulation, and affects cellular functionality by altering retrograde signals. Aging, cancer, and many diseases are associated with heteroplasmy. In adult life, compared to early life, there is a downregulation of mitochondrial genes (increasing the likelihood of mitochondrial mutations) and an upregulation of genes associated with the innate immune response, the response to proteotoxicity, the response to oxidative stress, and purine biosynthesis. Compromised mitochondrial function is associated with increased ROS production, increased mitochondrial DNA fragments, and other mitochondrial peptides that activate inflammatory responses. Mitochondrial genomes do not recombine or only recombine very little (Tower, 2015; Maude et al., 2019). The question arises if mtDNA mutations will increase in frequency through the effect of human migration. If so, we can expect an increase in the number of people suffering from mitochondrial diseases caused by those mutations. In order to prevent this process, it is necessary to investigate the way mtDNA mutations are selected and which can or cannot be related to disturbances in OXPHOS (Stewart and Chinnery, 2015).

### 3.2 Aerobic glycolysis and chronic disease

Modern life puts a real burden on the normal functioning of mitochondria in multiple organs. Factors such as sitting time (Nogueira Silva Lima et al., 2021), high-calorie diet (Nogueira Silva Lima et al., 2021), sleep disturbance (Saner et al., 2021), and alcohol abuse (Wang et al., 2010) and factors that are hardly evitable such as environmental pollution (Grytting et al., 2022), light pollution (Grytting et al., 2022) diesel exhaust and the use of multiple medicines, including NAISDs (Tang et al., 2022), can produce a state of low-grade inflammation (LGI) associated with many chronic diseases (de Punder and Pruimboom, 2015a). LGI and mitochondrial dysfunction are two cross-connected mechanisms. Although mitochondrial dysfunction can induce low-grade inflammation (Zampino et al., 2020), LGI can cause mitochondrial dysfunction (Schmitt and Gaspar, 2022). OXPHOS defects involve energetic cost, hypermetabolism, and increased aging velocity in most, if not all, organs (Diaz-Vegas et al., 2020; Sturm et al., 2023). Mitochondria-induced inflammation can be highly detrimental to overall health. Therefore, several control mechanisms exist to prevent mitochondria-driven inflammation and mitochondria from becoming damage-associated molecular patterns (DAMPs) and even prevent autoreactivity and possible autoimmune diseases (Marchi et al., 2022).

The Baltimore Longitudinal Study of Aging, investigating a total number of 669 individuals with an average age of 67 years, showed that participants with lower mitochondrial oxidative capacity exhibited hallmarks of inflammation, specifically showing markedly higher levels of interleukin-6 and C-reactive protein, as well as increased erythrocyte sedimentation rate compared with participants with better oxidative capacity, independent of age and sex (Zampino et al., 2020). The authors of this study proposed that products of damaged mitochondria activate the immune system, which in turn causes “inflammaging,” whereas oxidative species cause inflammation and detrimental aging. The possibility of mitochondrial damage should be considered part of the fact that organisms live. Therefore, it seems logical that many mechanisms are in place to prevent mitochondrial damage and the subsequent possibility of systemic inflammation. Marchi et al. described the different protective pathways of mitochondria-induced inflammation, including mitophagy, autophagy, and cell apoptosis (Zampino et al., 2020; Marchi et al., 2022; Marchi et al., 2023). Although mitochondria-induced inflammation has been evidenced in multiple chronic diseases, interventions are scarce, and only one medicine, venetoclax, has been approved till now (Roca-Portoles et al., 2020; Olivas-Aguirre et al., 2021).

This review proposes that the use of physiological stress triggers, such as intermittent fasting, intermittent cold, and intermittent hypoxia, should be able to provide primary and secondary prevention of mitochondrial-induced systemic inflammation (the scope of this review).

As aforementioned, LGI also produces multiple metabolic disturbances in different organs through the switch from OXPHOS to aerobic glycolysis, firstly in the immune system (Pålsson-McDermott and O’Neill, 2020) and secondly in other organs through long-term insulin resistance and hyperglycemia (Guzmán-Ruiz et al., 2014).

Aerobic glycolysis in non-immune cells is responsible for the production of biomass. On the one hand, site aerobic glycolysis can protect against oxidative stress and serve as an anabolic pathway of cell repair, growth, and cell division (Brand, 1997; Yuan et al., 2019; Bell et al., 2020; Takahashi, 2021). On the other hand, long-term aerobic glycolysis, called the Warburg effect, leads to cell swelling, metabolic disturbances, lack of ATP, and, depending on the type of cell, cell death through apoptosis or necrosis and possibly cancer (Schwartz et al., 2017; Cassim et al., 2020). Modern life is responsible for the metabolic switch from OXPHOS to aerobic glycolysis through LGI, energy abundance caused by a high-calorie diet, lack of physical activity and sitting time, leading to chronic hyperglycemia and a surplus of fatty acids (Segovia et al., 2014; Barrea et al., 2017; Cuevas-Sierra et al., 2019; Milanova et al., 2019; Nicolaidis, 2019; Setayesh et al., 2019; Khan et al., 2021a; Drozdz et al., 2021; Keipert and Ost, 2021). We speculate that aerobic glycolysis in non-dividing and dividing cells should be considered the central pathway of most, if not all, chronic diseases, including most types of cancer (and its hallmarks) (Kaur et al., 2020; Kaur et al., 2022), neurodegenerative diseases, such as Alzheimer (Traxler et al., 2022), and cardiovascular diseases (Tran and Wang, 2019).

Long-term aerobic glycolysis can uncouple enzymes of normal OXPHOS, causing a chronic Warburg effect and accumulation of intracellular fatty acids, nucleotides, amino acids, and, through the activation of the polyol-mechanism, sorbitol and sorbitol-attracted water (Singh et al., 2021; Tigchelaar et al., 2022). Chronic accumulation of biomass through aerobic glycolysis and the polyol pathway is responsible for retinopathy, nephropathy, and neuropathy next to all aforementioned diseases (Balestri et al., 2022)

In line with the reviewed data, people suffering from metabolic syndrome (Mets) are susceptible to the development of many, if not all, CNCDs. MetS is characterized by numerous metabolic dysregulations, including insulin resistance, leptin resistance, dysregulated hypothalamus—hypophysis—suprarenal gland axis function, atherogenic dyslipidemia, vascular calcification, central obesity, mitochondrial dysfunction, and altered blood pressure. All these disrupted mechanisms lead to immune dysregulation and LGI, which are part of the hallmarks responsible for the development of CNCDs (Uzunlulu et al., 2016; Pruimboom and Muskiet, 2018; Drozdz et al., 2021; Khorshidian et al., 2021; Muriel et al., 2021; Fahed et al., 2022).

Many of the pathophysiological mechanisms that characterize MetS are related to mitochondrial dysfunction caused by excessive ROS production when suffering from metabolic disturbances. The oxidant/antioxidant gradient normally protects against oxidative damage. In case of oxidative pressure that exceeds anti-oxidative capacity, mitochondria and other organelles can be severely damaged. Several mechanisms and risk factors can lead to oxidative overload (Tian et al., 2022), including the upregulation of fatty acid oxidation that increases NADH and FADH2 load. Spilling some of these high-energy electrons off the TCA chain leads to excessive ROS production. The excessive amounts of glucose and free fatty acids in adipocytes activate NADPH oxidase, an enzyme that produces H2O2 (Annie-Mathew et al., 2021; Fahed et al., 2022; Wallace, 2015). Insulin-resistance induces an increase in the production of oxidative stress, reduced OXPHOS, and energy production (Wu et al., 2005; Oliveira et al., 2014; Navarro-Ledesma et al., 2022). A sedentary lifestyle reduces mitochondrial density, whereas mitochondrial biogenesis only functions in response to high-energy requirements (Magalhães et al., 2013; Pruimboom et al., 2016). The absence of a healthy dietary pattern (diversity in vegetables and spices, mushrooms, good healthy proteins and fats, fruits), which we often find in people suffering from MetS, leads to deficiency in a variety of polyphenols and micronutrients (Phillips et al., 2019).

Polyphenols are a group of substances with evidenced hormetic effects in healthy food. They are phytochemical compounds that improve metabolism, cell signaling, and mitochondrial health; the phenolic compound works as a hormetic trigger. The same mechanism characterizes the impact of the use of acute physiological stress strategies, such as intermittent fasting, therapeutic cold, therapeutic heat, and intermittent hypoxia. We speculate that the absence of hormetic triggers in modern life will decrease the transcription of NRF1 and NRF2, subsequently TFAM and mitochondrial transcription factor B2 (TFB2M), and ultimately leading to a decrease in mitochondrial mass (Kokura et al., 2007; Harvie and Howell, 2016; Chung et al., 2017; Alavi et al., 2021; Parsamanesh et al., 2021; Zeraattalab-Motlagh et al., 2021; Lavallee et al., 2022; Pak et al., 2022). The absence of hormetic triggers can also alter the activity of PGC-1α, which could result in metabolic dysfunction of tissues, leading to the development of various metabolic diseases (Vandenbeek et al., 2018).

### 3.3 The impact of modern life on the Warburg effect and mitochondrial dysfunction

**FIGURE 3:**
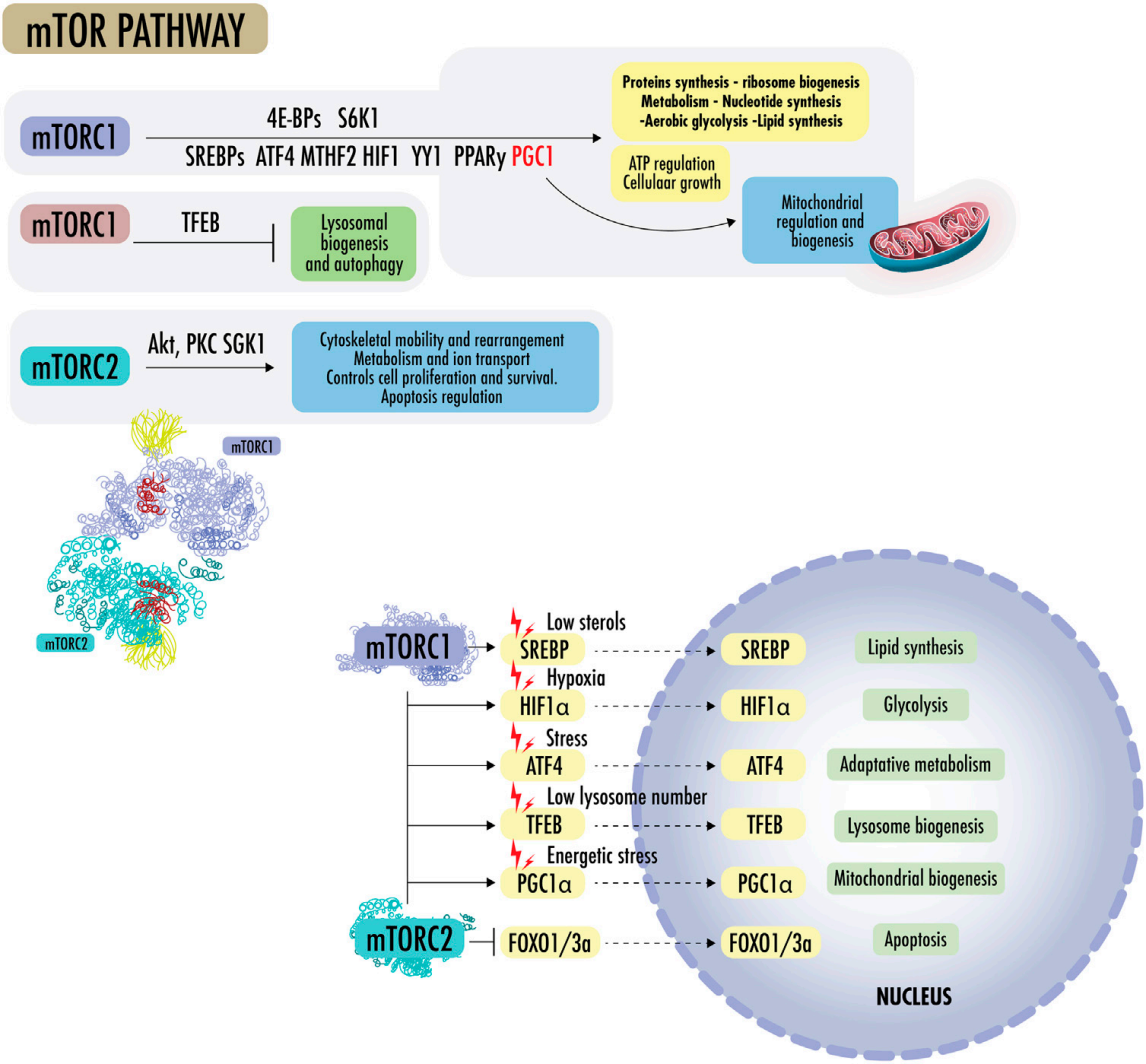
mTOR pathway: mTORC1 promotes protein synthesis by phosphorylating eukaryotic initiation factor 4E-binding proteins (4E-BPs) and p70 S6 kinase 1 (S6K1), increasing the production of ATP, nucleotides, and lipids. The lipid synthesis occurs primarily through the sterol regulatory element, protein-binding protein ½ (SREBP1/2), and peroxisome proliferator-activated receptor-γ (PPARγ). In the absence of sterols, SREBPs translocate to the nucleus to regulate genes for *de novo* cholesterol and other lipid synthesis. mTOR1 regulates the supply of one-carbon units for the biosynthesis of nucleotides for DNA/RNA replication, as it regulates methylenetetrahydrofolate dehydrogenase 2 (MTHFD2), the mitochondrial tetrahydrofolate cycle enzyme, carbamoyl-phosphate synthetase 2 (CPS2), aspartate transcarboxylase (ATCase), dihydroorotase (CAD), and the rate-limiting enzyme in pyrimidine biosynthesis, among others. mTORC1 regulates the transcription factor hypoxia-inducible factor 1α (HIF1α), which increases the expression of glycolytic enzymes and favors glycolysis over OXPHOS. mTORC1 activates mitochondrial transcripts through 4E-BP1 and stimulates mitochondrial biogenesis by driving PGC1α. mTORC1 can simultaneously activate SREBPs, transcription factor ATF4, HIF1, Yin Yang 1 (YY1), PPARy, and PGC1a to drive mitochondrial regulation, ATP regulation, macromolecules synthesis, and cellular growth, blocking lysosomal biogenesis through transcription factor EB (TFEB). mTORC2 effectors include protein kinase B (Akt/PKB) and protein kinase C alpha (PKCα), a member of the AGC family of protein kinases (PKA/PKG/PKC). mTOR2 plays a role in cytoskeletal rearrangement, actin regulation, chemotaxis, migration, and cell survival. Akt is a central early effector in the phosphatidylinositol 3-kinase-protein kinase B (PI3K) pathway, where it mediates the cellular response to insulin and promotes proliferation. Akt regulates metabolism to resist stressors through the transcription factors of FOXO1/3a and NAD kinase. In addition, Akt can mediate between mTORC1 and mTORC2 complexes by inactivating tuberous sclerosis complex 2 (TSC2), a strong inhibitor of mTORC1 activity.

Figure 3 States of nutrient abundance or nutrient deprivation are sensed and regulated by the “nutrient signaling” pathway, the growth axis, and regulation mechanisms of growth and division. The mechanistic target of rapamycin (mTOR) is seen as a central hub of nutrient signaling, cell growth, and division. It facilitates the metabolic switch between the use of OXPHOS and aerobic glycolysis (Liu and Sabatini, 2020a). It is also seen as a regulator of mitochondrial functions and is influenceable by acute stressors; therefore, it is of special interest in this review. mTOR controls biomass accumulation and metabolism by modulating key cellular processes, including protein synthesis and autophagy. mTOR is a 289-kDa serine/threonine protein kinase in the PI3K-related protein kinase (PIKK) family. It is made up of two complexes called mTORC1 and mTORC2. On cellular metabolic demand, mTORC1 and mTORC2 initiate biosynthetic cascades to support cell proliferation and anabolic state (Blagosklonny, 2013),

mTORC1 promotes protein synthesis by phosphorylating eukaryotic initiation factor 4E-binding proteins (4E-BPs) and p70 S6 kinase 1 (S6K1), increasing the production of ATP, nucleotides, and lipids. The lipid synthesis occurs primarily through the sterol regulatory element, protein-binding protein ½ (SREBP1/2), and peroxisome proliferator-activated receptor-γ (PPARγ). In the absence of sterols, SREBPs translocate to the nucleus and regulate genes for *de novo* cholesterol and other lipid synthesis. mTOR1 regulates the supply of one-carbon units for the biosynthesis of nucleotides for DNA/RNA replication. It further regulates methylenetetrahydrofolate dehydrogenase 2 (MTHFD2), the mitochondrial tetrahydrofolate cycle enzyme, carbamoyl-phosphate synthetase 2 (CPS2), aspartate transcarboxylase (ATCase), dihydroorotase (CAD, the rate-limiting enzyme in pyrimidine biosynthesis), among others. mTORC1 regulates the transcription factor hypoxia-inducible factor 1α (HIF1α), which increases the expression of glycolytic enzymes and favors glycolysis over OXPHOS. mTORC1 activates mitochondrial transcripts through 4E-BP1 and stimulates mitochondrial biogenesis by driving PGC1α (Summer et al., 2019). mTORC1 can simultaneously activate SREBPs, transcription factor ATF4, HIF1, Yin Yang 1 (YY1), PPARy, and PGC1a to drive mitochondrial regulation, ATP regulation, macromolecule synthesis, and cellular growth, blocking lysosomal biogenesis through transcription factor EB (TFEB) (Liu and Sabatini, 2020b; Zhu et al., 2022b; Khan et al., 2022; Cui et al., 2023).

mTORC2 effectors include protein kinase B (Akt/PKB) and protein kinase C alpha (PKCα), which is a member of the AGC family of protein kinases (PKA/PKG/PKC). mTOR2 plays a role in cytoskeletal rearrangement, actin regulation, chemotaxis, migration, and cell survival. mTORC2 also collaborates with PDK1 to activate other AGC family kinases and the Akt oncogene. Akt is a central early effector in the phosphatidylinositol 3-kinase-protein kinase B (PI3K) pathway, where it mediates the cellular insulin response and promotes proliferation. Akt regulates metabolism to resist stressors through the transcription factors of FOXO1/3a and NAD kinase. In addition, Akt can mediate between mTORC1 and mTORC2 complexes by inactivating tuberous sclerosis complex 2 (TSC2), a strong inhibitor of mTORC1 activity, and phosphorylating mSin1, an obligate component of mTORC2. As the FOXO proteins are regulated by SGK1 and Akt, SGK can be the mTORC2 effector, whereas Akt appears to be a more subtle modulator (Liu and Sabatini, 2020a; Choi et al., 2020).

Macrophages exposed to an inflammatory stimulus switch their metabolism from OXPHOS to aerobic glycolysis, increasing glucose metabolism enzymes, activating transcription factors, such as mTOR and HIF1α, to support ATP production independently of the availability of oxygen and facilitating the synthesis of nucleotides, fatty acids, and proteins to support cellular function (Peruzzotti-Jametti et al., 2021). In the cells of the immune system, maintained glycolysis tends to switch immune cells to a pro-inflammatory phenotype. Upregulated glycolysis is observed in many immune cells, such as T cells, NK cells, B lymphocytes, and dendritic cells. Upregulated glycolysis could lead to immune activation with altered antibody production, lower self-tolerance, and increased cytokine release, resulting in transcriptional and post-transcriptional upregulated pro-inflammatory mediators affecting immune efficiency. This also leads to increased post-prandial inflammation responses, an important risk factor for LGI and its consequences (Teng et al., 2019; Kornberg, 2020).

In the situation of substrate surplus, adipose tissue also switches from OXPHOS to Warburg/glycolysis developing hypertrophy (increased adipocyte size), hyperplasia (increased numbers of adipocytes), or both (Choe et al., 2016). Hypertrophic adipose tissue is associated with immune cell recruitment, increased basal fatty acid release, pro-inflammatory cytokine release, hypoxia, necrotic-like abnormalities, fibrosis, decreased adiponectin, impaired insulin sensitivity, and insulin-dependent glucose uptake related to a defect in GLUT4 trafficking (Choe et al., 2016). The differentially hyperplasic adipose tissue shows increased adiponectin, decreased basal fatty acid release, and improved insulin sensitivity. It also releases pro-inflammatory cytokine and induces hypoxia and fibrosis but with fewer immune cells and a higher proportion of small adipocytes, leading to a healthier adipose tissue phenotype (Choe et al., 2016).

The adipocytes that determine a pro-inflammatory immune system are white adipocytes with low mitochondrial density. Beige and brown adipocytes characterized by high healthy mitochondrial density are metabolically efficient and maintain an anti-inflammatory and antitumoral phenotype (Corrêa et al., 2019; Symonds et al., 2019; Zoico et al., 2019). In particular, white hypertrophic adipose tissue acts as an endocrine regulator that uses many adipokines such as hormones (leptin and adiponectin); some peptides such as resistin, angiotensinogen, and apelin, among others; immune factors; and inflammatory cytokines such as interleukin-1, interleukin-4, interleukin-6, interleukin-8, interleukin-10, interleukin-18, monocyte chemoattractant protein-1, nerve growth factor, neuropeptide Y, retinol-binding protein-4, transforming growth factor-β, tumor necrosis factor- (TNF-) alpha, vascular endothelial growth factor (VEGF), visfatin, omentin, and chemerin. All of these oversee controlling orexigenic (hunger) or anorexigenic (satiety) stimuli (Coelho et al., 2013; Szewczyk-Golec et al., 2015; Tsatsanis et al., 2015; Katsiki et al., 2020)

Risk factors of Mets and switchers to glycolysis are excess sugar and unhealthy fats consumption, obesogens, sedentarism, consumption of processed food, higher intake of fructose (corn syrup, juices, soft drinks, and sweets), and many other processed foods (Dornas et al., 2015; Khorshidian et al., 2021; Muriel et al., 2021; Coronati et al., 2022; Muskiet et al., 2022). Moreover, pollution and exposure to obesogens such as endocrine-disrupting chemicals (EDC) will increase the fat amount, inflammation, and adipocyte dysfunction (Uzunlulu et al., 2016; Pruimboom and Muskiet, 2018; Heindel and Blumberg, 2019; Dietert, 2021; Khorshidian et al., 2021; Mohajer et al., 2021; Muriel et al., 2021).

Other less cited, modern factors are involved. Loneliness and chronic social isolation are also associated with upregulated lipid synthesis and a metabolic switch from OXPHOS to aerobic glycolysis and glycolytic pathway gene expression (Williams et al., 2009). Social interactions should be considered a basic need, just as other human needs, such as eating, breathing, and sleeping (Shen et al., 2022b).

The benefits of social interaction include better mental health, improved sleep quality, increased life expectancy, and improved immunological and metabolic health (Xiong et al., 2022). Related to metabolic changes observed in loneliness or chronic isolation, HPA axis chronic activation leads to the elevated secretion of cortisol, increased blood glucose, glycogenolysis, and insulin resistance that ends up in engaging in unhealthy habits and decreasing satiety signaling. A recent 20-year follow-up study including 24,024 participants found that loneliness was associated with a twofold risk of type 2 diabetes in participants who informed “very much” on experiencing loneliness than those who had not felt lonely (Henriksen et al., 2022).

New insights into the way mitochondria respond to social behavior include mitochondria-derived stress mediators (steroid hormones produced by mitochondria) and blood mitokines. An emerging circulating mitokine is cell-free mtDNA (cf-mtDNA) present in human blood, which activates immune receptors and triggers inflammatory responses. Acute psychological stress (Trumpff et al., 2019), major depression (Lindqvist et al., 2018), and intense physical activity (Stawski et al., 2017) modify mitokines production and increase circulating cf-mtDNA. Interdisciplinary approaches that involve mitochondrial signaling in resilience, aging, and metabolism are needed, and perhaps mitochondria should be defined as social organelles (Picard and Sandi, 2021).

Not only should the modern lifestyle be considered toxic for cell and mitochondrial functioning, but also medication can induce mitochondrial toxicity and a metabolic switch to glycolysis because of the impairment of OXPHOS. Many drugs have been reported to cause mitochondrial harm and damage, including benfluorex, rosiglitazone, celecoxib, ponatinib, etoricoxib, diclofenac, and remdesivir (Tang et al., 2022). Oncological drugs are reported to cause structural damage to mitochondria, including downregulated ferroptosis, accumulation of lipid peroxides, mitochondrial swelling, cristae disappearance, and matrix cavitation, as found in research with the oncological medicine doxorubicin (DOX) (Tadokoro et al., 2020; Tang et al., 2022). Related to mitochondrial complexes, zoniporide, naproxen, dronedarone, and mubritinib inhibit complex I (Tang et al., 2022). Complex II is compromised by propranolol and atenolol. Celecoxib suppresses complex IV, and As_2_O_3_ inhibits complexes I, III, and IV. Non-steroidal anti-inflammatory drugs (NSAIDs), such as nimesulide, meloxicam, and acetylsalicylate, also o inhibit OXPHOS.

Lipophilic drugs can damage phospholipids on the IMM, especially cardiolipin, or activate the mitochondrial permeability transition pore (mPTP) (Tang et al., 2022). Studies have demonstrated the presence of mitochondria-induced myopathies caused by reduced respiratory enzyme activity, calcium leakage, and oxidative stress in patients treated with statins in addition to rhabdomyolysis reported in 1 in 10,000 patients (Stoker et al., 2019; Xiang et al., 2021; Mima, 2022; Tang et al., 2022). Cancer radiation therapy in animal models induces aerobic glycolysis through ROS (Zhong et al., 2013). Chemical cancer therapies, antiviral or antiretroviral drugs, antibiotics, antidiabetic drugs, non-steroidal anti-inflammatory agents, anesthetics, and many others, impair healthy mitochondrial function altering the metabolism of many cell types, including those of the immune system (Stoker et al., 2019; Tang et al., 2022).

Almost all mentioned risk factors leading to the Warburg effect and long-term aerobic glycolysis are characterized by high blood glucose levels and an abundance of free fatty acids. The use of hormetic triggers could serve as an antidote against modern life because of a rerouting of cellular metabolism from cytosolic glycolysis to mitochondrial OXPHOS (Bianchi et al., 2015; Ng et al., 2022; Pak et al., 2022).

Lifestyle interventions, such as avoiding sedentarism, improving social connection, avoiding mitochondrial toxins/drugs (if possible), and lowering glucose intake, combined with fasting targets, could serve as an antidote against mTOR aging effects and glycolytic reprogramming. Intermittent fasting alone already induces an anti-Warburg effect (Bianchi et al., 2015; Choi et al., 2016; Mattson et al., 2017; de Cabo and Mattson, 2019; Kornberg, 2020; Locati et al., 2020; Cuevas-Cervera et al., 2022) (elaborated in PART III).

### 3.4 The focus on cancer as a “Warburg” disease

Cancer is considered a disease characterized by hallmarks such as aerobic glycolysis in most, if not all types of, cancers. Cancer manipulates its own metabolism and the metabolism of cells surrounding a tumor, making it a selfish-metabolic disease (Vaupel and Multhoff, 2021). Therefore, not only does the tumor cell itself depend on aerobic glycolysis for the initiation and progression of cancer, but also the tumor activates the Warburg effect in cells of the tumor microenvironment (TME), including immune cells and cancer-affected fibroblasts. By doing so, the tumors cells create a hyper-acidic, nutrient-deficient environment combined with changes in glutamine load, fatty acid metabolism, and hypoxic states that support tumor aggressivity and growth (Correnti et al., 2022; Zhu et al., 2022c; Salita et al., 2022; Wang and Abolhassani, 2022; Mora Barthelmess et al., 2023)

Different from what was earlier assumed, the acceleration of aerobic glycolysis is not a consequence of dysfunctional mitochondria perse and a compensation for the poor ATP yield per molecule of glucose. Instead, in most tumors, the Warburg effect is an essential part of selfish metabolic reprogramming. As discussed earlier, mitochondria play an important role in cell fate. The exclusion of mitochondria from the metabolism prevents the cell and its cancer from being killed by the cell fate mechanisms of mitochondria. The glycolytic switch is an early event in oncogenesis and primarily supports cell survival (Vaupel and Multhoff, 2021). The metabolic transformation leading to the Warburg effect we observe in cancer also underlies neuronal degeneration in sporadic AD (Traxler et al., 2022). Strategies intervening in this metabolic switch, inhibiting glycolysis and glutaminolysis, and promoting OXPHOS—keeping mitochondria healthy—could be interesting strategies in the fight against these conditions and others related to the Warburg effect (Manzi et al., 2015; Branco et al., 2016; Smyl, 2016; Cusso et al., 2019; Tran et al., 2020).

Besides preventing pro-apoptotic pathways mediated by mitochondria, aerobic glycolysis enables a list of other malignant progression and survival advantages for cancer cells. Examples are accelerated glycolytic fluxes, ATP generation, a backup and diversion of glycolytic intermediates, the biosynthesis of nucleotides, the production of non-essential amino acids, lipids and hexosamines, maintenance of cellular redox homeostasis, low ROS formation, inhibition of pyruvate entry into mitochondria, lactate accumulation, stimulating sustained proliferation and suppression of anti-tumor immunity, and extracellular acidosis, which accelerates malignant progression and drives resistance to conventional therapies (Vaupel and Multhoff, 2021). As the metabolic shift seems an important stone early in the domino effect of cancer initiation, preventing or intervening in this switch seems an important intervention for primary and secondary prevention of cancer.

The Warburg effect results from an interplay of different mechanisms and driving processes. HIF1 overexpression, oncogene activation (cMyc, K-ras- mTORC1, and Akt), activation of signaling pathways (PI3K/Akt/mTORC1, Ras/Raf/MEK/ERK), an increase in glucose (GLUT) and lactate (MCT4) transporters, and the activation of glycolytic enzymes (HK2, PFK1, ENO1, PKM2, and LDHA) (Wallace, 2005; Choudhury et al., 2020; Han et al., 2021; Mariani et al., 2021; Pluimakers et al., 2021; Shen et al., 2021; Vaupel and Multhoff, 2021; Zhu et al., 2022c) are all part of the metabolic change observed in cancer cells. Furthermore, the functions of tumor suppressors (mutant p53, mutant PTEN, and microRNAs 29, 143, and 144), Sirtuins 3 and 6, and the AMPK signaling pathway (Kumar et al., 2016; Alhebshi et al., 2021; Jazvinšćak Jembrek et al., 2021; Zhang et al., 2022a) are inhibited to prevent cell death and reduce metabolic stress of tumor cells. The metabolic changes belonging to the Warburg effect can be influenced by the known physiological hormetic triggers that could serve as primary and perhaps secondary preventive interventions (Jazvinšćak Jembrek et al., 2021). For instance, intermittent fasting programs could perhaps serve as Warburg antidote, blocking enzymatic pathways and creating amino-acid and glucose starvation (Lee et al., 2012; Harvie and Howell, 2016; Bauersfeld et al., 2018; Fassier et al., 2018; Lende et al., 2019; Tiwari et al., 2022). Glutamine executes multiple functions in cancer cells. Besides being an energy source, glutamine is a so-called anaplerotic molecule. Glutamine can replenish the TCA cycle with intermediates extracted for biosynthesis. In this regard, glutamine is an alternative source for the TCA cycle. Thereby, glutamine uptake by the energetically transformed cell contributes to the formation of nucleotides and fatty acids and has an important role in the homeostasis of ROS (Alhayaza et al., 2020). Another important oncometabolite is leucine, which, together with glutamine, can activate the mTOR complex (cell growth master) (Scalise et al., 2017b). As aforementioned, intermittent fasting could serve as an mTOR antidote.

#### 3.4.1 A brief description of glutaminolysis in cancer

When glutamine is absorbed by metabolically transformed cells, it is converted into glutamate. Thereafter, glutamate is converted into α-ketoglutarate, which enters the TCA in the mitochondria, where the reaction is catalyzed by succinyl-CoA synthetase with the resulting production of ATP. In the above process, one of the five carbon atoms of glutamine is released as CO_2_. The remaining four carbon atoms of glutamine are exported to the cytosol as malate, which, in turn, can give rise to different metabolic pathways useful for cancer cells, including the conversion into pyruvate (Scalise et al., 2017c). Pyruvate can be converted into lactate for aerobic glycolysis and ATP production. The conversion of glutamine into glutamate is regulated by an oxidative reaction orchestrated by glutaminase (Zhang et al., 2022b). Alternatively, malate can enter the TCA cycle as a molecule with four carbon atoms, including asparagine alanine-serine cysteine-preferring transporter 2 (ASCT2) substrates (Scalise et al., 2018). Malate is converted into oxaloacetate via malate dehydrogenase and then into aspartate via aspartate aminotransferase (Scalise et al., 2018). The alternative output substrate of ASCT2, serine, can be derived from glucose (via phosphoglycerate dehydrogenase/phosphoserine aminotransferase/phosphoserine phosphatase). The enzymes mentioned serve as important targets in the development of anticancer therapies. The glutaminase enzyme is produced by two different genes: GLS1 and GLS2 (Scalise et al., 2017b; Zhang et al., 2022b). These genes are important therapeutic targets, and inhibition could serve as a promising cancer intervention.

Other targets of cancer preventive interventions are glucose and glutamine transporters and their increase in the cancer process. Cancer cells overexpress glucose transporters (GLUTs), sodium-dependent amino acid transporters such as ASCT2, and sodium-independent amino acid transporters for signaling, such as LAT1 (Scalise et al., 2017c; Scalise et al., 2020). Glutamine is also recognized at the plasma membrane by SLC receptors (including members, e.g., SLC1, SLC6, SLC7, SLC36, and SLC38). Within this family, the major and best-characterized glutamine transporter is SLC1A5. SLC1A5 is currently known as ASCT based on preliminary observations of substrate specificity, although the actually preferred substrate is now known to be glutamine (Scalise et al., 2017c). The most likely exchanged amino acids by SLC1A5 are asparagine, threonine, or serine, and their transport, together with glutamine, allows the entry of 1–2 carbon atoms into the cell, which can then be oxidized in the TCA with ATP production in the mitochondria. The increase in the plasmatic concentration of serine and threonine is well-described in cancer (Scalise et al., 2017c). It seems clear that amino acids are involved in cancer metabolic rewiring, and several are essential for the initiation and progress of cancer (Scalise et al., 2020; Wang et al., 2022a; Shen et al., 2022c)

As touched upon briefly, the metabolic transformation seen in tumor cells also enhances a favorable ROS environment. ROS accumulation can directly affect DNA integrity, and ROS-mediated DNA damage could favor the initiation stage of tumorigenesis. ROS have also been associated with epigenetic alterations that favor oncogenic transformation. ROS-induced hypermethylation of the promoter region of tumor suppressor genes has been shown to promote carcinogenesis. Cancer cells also need to keep ROS production under control, and glutamine converted in glutamate serves the synthesis of glutathione peroxidase as a major anti-oxidative enzyme from glutamine. Intervening in this pathway also seems important (Shi et al., 2022).

Another possible factor causing the metabolic transformation of cells into tumor cells is related to the presence of mutations in mitochondria caused by “modern life.” Enzymes produced by mutated mtDNA are related to cancer development and the use and production of several oncometabolites (Kes et al., 2020). Oncometabolites are signaling molecules derived from mitochondria dysfunction. Some examples found in various types of cancer are loss-of-function mutations of the iron-sulfur B subunit of the succinate dehydrogenase complex (SDHB) and fumarate hydratase (FH) with increased levels of fumarate and/or succinate (Haas and Nathanson, 2014; Gupta et al., 2019). Others are gain-of-function mutations in cytosolic and mitochondrial isocitrate dehydrogenase (IDH) isoforms 1 and 2 with the production of 2-hydroxyglutarate (2-HG). These combinations lead to the loss of α-ketoglutarate (α-KG) production and simultaneous gain of 2-HG that alters transcriptional patterns of histone methylation regulation and whole DNA methylation in favor of tumor growth (Cassim et al., 2020).

Pyruvate kinase M (PKM) is the glycolytic enzyme that converts phosphoenolpyruvate to pyruvate. The PKM gene codes for two isoforms, PKM1 and PKM2, which code for 22 different amino acids. PKM2 is the most common isoform of this enzyme in cancer (van Niekerk and Engelbrecht, 2018; Li et al., 2022a). The pathological isoform of PKM toward the cancer-associated PKM2 isoform causes metabolic and transcriptional changes. These alterations occur through the lack of metabolic activity of PKM2. PKM2 specifically interacts with the STAT3 and HIF1a transcription factors and enhances them, together exerting pro-oncogenic programs in which HIF1a is aberrantly activated despite a normoxic environment (Traxler et al., 2022).

Altogether, pro-carcinogenic changes have a solid metabolic basis, and changes in metabolism are a leading therapeutic target for the treatment of patients with cancer, which is among the leading causes of mortality and, in many countries, risk factor number one (Bray et al., 2018; Bray et al., 2021). As many processes are involved at the same time, primary and perhaps secondary prevention should be achieved by multiple lifestyle interventions. In the scope of this review, the use of physiological hormetic triggers possibly stops the domino cascade, leading to the development of neoplasms.

### 3.5 Infection and endotoxemia

Most CNCDs involve endotoxemia, LGI, and/or intestinal permeability. Intestinal barrier dysfunction results from food poisoning, dietary factors, and dysbiosis, and increased permeability leads to the translocation of sterile toxins and living or dormant microbes and lipopolysaccharides (LPS) into the bloodstream (de Punder and Pruimboom, 2013; de Punder and Pruimboom, 2015b).

The resulting endotoxemia leads to a sustained or low inflammatory and stress response. Stress induces corticotropin-releasing hormone (CRH), which stimulates mucosal cell cytokine release, which can, in turn, increase TNF that results in loose tight junctions and pathological gut permeability (Takakura and Pimentel, 2020; Michielan and D’Incà, 2015; Fasano, 2020; Woodhouse et al., 2018; Cristofori et al., 2021). Translocation of intestinal xenotoxins and micro-organisms should be considered one of the main pathways leading to low-grade inflammation (Laugerette et al., 2011; de Vries et al., 2014; Mohammad and Thiemermann, 2021). In the intestine only, there are more than 100 billion microbes, including archaea, bacteria, fungi, protozoa/parasites, and viruses. The immune, mitochondrial, and metabolic characteristics of the gut environment will depend on the enterotype (distribution taxonomic classification of microbial families, mucus quality, and diversity of each intestine) (Pimentel and Lembo, 2020). A gut pathological microbiome has been related to chronic disease, LGI, and mitochondrial dysfunction, and the so-called atopbiome in other tissues is frequently recognized as a risk factor for chronic diseases. Our industrialized society, with the presence of a wide range of toxic chemicals, metals, and antibiotics, has completely changed the microbiome, which affects mitochondrial functionality and organ function (Demeneix, 2019; Martel et al., 2022).

Modern life also comes with environmental exposure to many pathogenic microorganisms and resistant microbes exposed to insecticides or pesticides. Dysbiosis caused by pathobionts impairs mitochondrial integrity through multiple pathways, impairing healing mechanisms and promoting chronicity. Therefore, improving mitochondrial health is a way to prevent ROS overload and mtDNA deterioration and subsequently improve immune responses (Michielan and D’Incà, 2015; Fasano, 2020; Martel et al., 2022; Akdis, 2021; Rizzetto et al., 2018; Chopyk and Grakoui, 2020; Mou et al., 2022). In other words, strengthening mitochondria as much as possible helps us deal with the deteriorating consequences of modern life.

An acute infection induces dysregulation in mitochondria-nuclear communication, which is part of the resolving response when it is time-restricted. In an LGI, this dysregulation lasts long and should be considered one of the most important steps in the development of chronic diseases (Sureshbabu et al., 2018; Sun et al., 2019; Piotrowicz et al., 2021). Mitochondria are a major location for the production of ROS, which is necessary to fight infections. Evolutionary pressure made pathogens exploit the mitochondrial influence in killing and developed mechanisms to disturb mitochondrial–nucleus communication in fighting infections and increased the survival of the struggling pathogens (Andrieux et al., 2021).

The existence and optimal functioning of pattern-recognizing receptors (PRRs) are essential for optimal immune functionality. There are four families of PRRs distributed throughout all types of cells in the human body, namely, TOLL-like receptors (TLRs), NOD-like receptors (NLRs), retinoic acid-inducible gene I-like receptors RIG-I (RLRs), RIG-I receptors, and C-type lectin receptors (CLR). Each PRR can trigger a response through the nuclear transcription factor Kappa beta (nfKb) that allows the transcription of interferon and pro-inflammatory cytokines dependent on the pathogen that activated the PRR (Sandhir et al., 2017; Andrieux et al., 2021).

In the antiviral response, the mitochondrial antiviral signaling protein (MAVS)—located in the outer membrane of the mitochondria—is involved in the recognition and detection of viruses via the RLR receptor signaling pathway. Mitochondrial dynamics (fusion/fission) also regulate the RLR signaling pathway. The interaction between MAVS and the mitochondrial outer membrane protein mitofusin (MFN) and mitochondrial fusion are required in the RLR signaling pathway. In animal models with the deletion of MFN1 and MFN2, responses via RLR are reduced and impair the antiviral response. ROS are essential in antimicrobial signaling and efficiency because macrophages and dendritic cells eliminate microorganisms by phagocytosis. The proximity between the alpha-phagosomes and the mitochondria allows mtROS to cross the phagosome and eliminate the pathogen (Sandhir et al., 2017; Tan et al., 2017).

The NRLP3-dependent inflammasome is also an immune response that induces inflammatory cell death (pyroptosis) and has the function of eliminating intracellular bacteria/viruses before they proliferate too much. It allows the recruitment of other immune cells to improve anti-infective efficiency. The activation of the NLRP3 inflammasome is related to the triggering of TLRs and the production of pro-IL1b and pro-IL18 via caspase-1 activation in response to microbial infection and cellular damage (PAMP and DAMP signaling). Free mtDNA, ROS, and the MAVS pathway are required for the recruitment of NLRP3 to mitochondrial membranes. The phospholipid cardiolipin translocates from the inner to the outer membrane of the mitochondria to bind to NLRP3 and promote its activation. The accumulation of mtDNA in the cytosol results in an antiviral immune response, and the oxidation of mtDNA and cardiolipin leads to the activation of the inflammasome that ultimately induces a pro-inflammatory response that should resolve in time (Kelley et al., 2019; Deo et al., 2020; Wang et al., 2022b; Harapas et al., 2022).

Besides the necessity of mitochondrial involvement in eliminating pathogens, damaged mitochondria can be the cause of inflammation due to their prokaryotic (bacterial) origin. Damage causes free mtDNA, which is perceived as DAMP, and triggers inflammation. Therefore, infection is not the only cause of inflammation; mitochondrial damage could be a cause as well (Tan et al., 2017; Kelley et al., 2019; Deo et al., 2020; Alfarouk et al., 2021; Andrieux et al., 2021; Harapas et al., 2022).

The role of mitochondria in the antibacterial response is evidenced by their role in apoptotic regulation. Host cells and their mitochondria produce ROS to damage the lipids, proteins, and nucleic acids of some bacteria. In addition, mitochondrial fission via dynamin-related protein 1 (DRP1) maintains cell homeostasis during infection to prevent propagation by modulating cell apoptosis because if the cell dies, the infection can spread (Andrieux et al., 2021; Yang et al., 2022).

Pathogen infection changes the mitochondrial- metabolic- and oxidative profile of cells. Infected cells cause dysregulation of several nuclear genes through retrograde signaling. When pathogens exploit and eliminate mitochondrial defense mechanisms, they are much more efficient in bypassing immune mechanisms. Pathogens have learned to use pathways that avoid both immune and mitochondrial anti-infective effects. The aberrant/persistent activation of the NLRP3 inflammasome leads to chronic inflammatory disorders with LGI and endotoxemia. The treatment of mitochondrial damage and gut permeability and the restoration of gut equilibrium are promising targets for optimizing and resolving infection at the time. Mitochondria are vital in triggering the immune response related to RLR, NLR, or TLR against viruses, protozoa, bacteria, fungi, or damage (Kelley et al., 2019; Curi et al., 2020; Saleh et al., 2020; Andrieux et al., 2021).

The persistence of pathological microbes harms the integrity of the intestinal barrier, mediating immune and mitochondrial failure. Even biomechanical disorders, such as intervertebral discopathies, seem to be associated with disc infection caused by oral and skin-derived pathogens (Rajasekaran et al., 2020; Li et al., 2022b; Shanmuganathan et al., 2022). Besides the aforementioned mechanisms, dysbiosis reduces the supply of calcium to the bone and impairs the ability to manufacture vitamins K2 and D and other important immuno-metabolic substances. Therefore, dysbiosis and the resulting endotoxemia affect the integrity of bones (Radaic and Kapila, 2021; D’Amelio and Sassi, 2018). Dysbiosis mediates more aggressive immunological disorders in genetically predisposed individuals, and dysbiosis is related to Multiple immune pathologies. Examples are multiple sclerosis (Opazo et al., 2018; Kinashi and Hase, 2021; Mou et al., 2022), type 1 diabetes (Lin and Zhang, 2017; Kinashi and Hase, 2021), inflammatory bowel diseases (de Oliveira et al., 2017; Lin and Zhang, 2017; Opazo et al., 2018), rheumatoid arthritis (Horta-Baas et al., 2017; Jethwa and Abraham, 2017; Lin and Zhang, 2017; Nikitakis et al., 2017), autoimmune liver diseases (Virili and Centanni, 2017; Chopyk and Grakoui, 2020), thyroid diseases (Knezevic et al., 2022), celiac disease (Cenit et al., 2015; Videhult and West, 2016), systemic lupus erythematosus (Opazo et al., 2018; De Luca and Shoenfeld, 2019; Konig, 2020), and atopic dermatitis and allergies (McKenzie et al., 2017; Shu et al., 2019; Carucci et al., 2021). The interconnection between endotoxemia, mitochondrial dysfunction, LGI, and even non-resolved infection demands more integrative interventions. Lifestyle interventions and the use of physiological hormetic stress triggers could serve as interventions for primary and secondary prevention of chronic diseases, the scope of this review.

### 3.6 Neurological diseases. Mitochondria in the brain

The metabolic activity of brain neurons is, together with the activity in the lungs and kidneys, the highest in the human body. Neurodegeneration and neurodegenerative diseases (Alzheimer’s, Parkinson’s, and ALS) or neuroinflammatory disorders (myalgia encephalitis/chronic fatigue syndrome (CFS), and chronic pain) are all related to mitochondrial dysfunction (Fairbrother-Browne et al., 2021; Kumar et al., 2022; Rey et al., 2022).

The disruption of any of the mitochondrial dynamics or alteration of functions, such as energy production, ROS production, calcium homeostasis, control of epigenetic and nuclear transcriptional processes, immune defense, lipid regulation, and glucose regulation, can have deleterious effects on neurons and neuroglial cells (Fairbrother-Browne et al., 2021; Kumar et al., 2022; Rey et al., 2022).

Although neurological diseases vary in terms of their underlying risk factors and mechanisms, mitochondrial dysfunction is common in most, if not all, of them. Mitochondria are necessary for the energy supply of neurological cells, as their energy consumption is incredibly high, with the brain as one of the most specialized and metabolically active organs. Accordingly, neurons in the brain have a dense population of mitochondria consuming 20% of the total energy expenditure, whereas the brain weight is only 2% of the total body mass. The human brain is dependent on a continuous demand for energy in the form of glucose that is converted into lactate by Glia cells because neurons use 80%–90% of energy in the form of lactate. In basal conditions, neurons and astrocytes will use the same amount of energy sources. Although neurons synthesize ATP through OXPHOS, astrocytes are specialized in metabolizing glucose through aerobic glycolysis, resulting in an enormous amount of lactate and pyruvate generation from glucose that can be transported to neurons by MCTs and hydrocarboxylic acid receptors 1 (HCAR1) (Sharma et al., 2019; Mani et al., 2021; Bhatti et al., 2022; Wang et al., 2022c; Zeviani and Viscomi, 2022).

Parkinson’s disease (PD) could be considered a mitochondrial dysfunctional disease. One of the mitochondrial mechanisms involved in PD is related to the function DJ-1 protein. DJ-1 is an oxidative stress sensor that prevents neuronal death induced by oxidative stress and inhibits the aggregation of α-synuclein via its chaperone activity. Dj-1 mutation causes mitochondrial dysfunction and accumulation of α-synuclein, the hallmark of PD (Dolgacheva et al., 2019; Chia et al., 2020). This could also lead to complex I deficiency in muscular and immune cells, substantia nigra, and platelets, as seen in patients with PD (Rey et al., 2022). MAM and ER defects induce translocation of the MAM components, such as IP3R, VDAC, and MFN1 and MFN12. This can lead to disturbed calcium homeostasis and cause misfolded proteins with impaired autophagy, distorted mitochondrial dynamics, and cell death (Sunanda et al., 2021). Sirtuins are NAD+-dependent protein deacetylases, and seven sirtuin members can be involved (SIRT1–SIRT7) (Grünewald et al., 2019; Lautrup et al., 2019). Alterations in mitochondrial-located sirtuins (SIRT3, SIRT4, and SIRT5) induce mitochondrial transcriptional problems, which might result in pathologies (He et al., 2022; Kumar et al., 2022). Iron is a key element for mitochondrial function and homeostasis, which is also crucial for the maintenance of the neuronal system. However, too much iron promotes oxidative stress, immune response, and altered mitochondrial proteins. Patients with PD show an over-storage of iron compared with controls that could be caused by blood barrier dysfunction and subsequently lead to iron storage in the substantia nigra (Cheng et al., 2022). Another explanation can be an upregulation of some iron-storage proteins such as mitoferritin, lactoferrin, and transferrin or increased expression of DMT1 in dopamine neurons and ceruloplasmin dysfunction, observed in multiple neurodegenerative diseases (Zhang et al., 2021a; Cheng et al., 2022).

In ALS, mitophagy and increased ROS are markedly involved in its pathogenesis, resulting in a reduced number of phagosomes at the neuromuscular junction (Tsitkanou et al., 2016; Baek et al., 2022; Cheng et al., 2022). Mutations (acquired or congenital) in the genes for the production of mitochondrial proteins FUS, TDP-43, SOD1, and C9ORF72 have been reported (Wong and Venkatachalam, 2019; Rey et al., 2022). More than 170 SOD1 mutations are associated with ALS. SOD1 codes for Cu-Zn superoxide dismutase, which is responsible for neutralizing superoxide radicals by catalyzing molecular oxygen and hydrogen peroxide. Mutations in FUS, TDP-43, SOD1, and C9ORF72 increase DNA damage, alter mitochondria fusion and fission, alter calcium homeostasis, and reduce the activity of respiratory chain complexes II and IV (Kodavati et al., 2020; Rey et al., 2022). This leads to structural abnormalities, such as swollen mitochondria, impaired MAM functions, augmented mitochondrial fragmentation, progressive loss of membrane potential, increased ROS production, and defective mitochondrial axonal transport (Kodavati et al., 2020; Rey et al., 2022). Inhibition of the action of the GTPase involved in mitochondrial fission dynamin-related protein 1 (DRP1) and reduced levels of mitochondrial metabolic proteins lead to many neurodegenerative diseases through the accumulation of misfolded TAU proteins called “tauopathies.” Normally, TAU is responsible for microtubule attachment and cytoskeleton stability in the brain (Petrozziello et al., 2022). Dysregulations of the cytoskeletal network, mitochondrial localization, and homeostasis are involved pathways in the initial steps for the development of neurodegeneration. In ALS, mitochondrial dysfunction increases mtROS and decreases ATP production. Dysregulation of mitochondrial proteins that impair fission, fusion, and mitophagy is responsible for disruptions of axonal transport and defects of the cytoskeletal organization (Petrozziello et al., 2022). Furthermore, mitochondrial dysfunction leads to impaired binding of motor proteins to microtubules, altered activities of kinases, and destabilization of the motor cargo binding. ALS further shows dysregulated PKN1 activity (Petrozziello et al., 2022). The serine/threonine-protein kinase N1 encoded in the *PKN1* gene is a regulator of synaptic transmission and maturation. PKN1 elevates excitatory amino acid transporter-3 (EAAT3) and other glutamate transporters. The resulting excitotoxic levels of glutamate disrupt the axonal trafficking of neurofilaments and may contribute to irreversible neurodegeneration (Petrozziello et al., 2022).

Mitochondrial dysfunction is also seen as a primary cause of AD. The brains of patients with AD show impaired glucose and oxygen metabolism and impaired activity of pyruvate dehydrogenase, ketoglutarate dehydrogenase, and cytochrome oxidase. Furthermore, mtDNA alterations, dysfunctional transmembrane amyloid beta, and tau protein accumulation are found, together with altered mitochondria morphology and respiratory chain dysfunction (Chen et al., 2021a; Castora et al., 2022; Rey et al., 2022).

Myalgic encephalitis (ME)/CFS are two other maladies associated with mitochondrial dysfunction. The two pathologies show common mechanisms with neuroinflammatory diseases, such as depression, fibromyalgia, and migraine. The co-occurrence of depression and pain is 30%–60% (Maletic and Raison, 2009). Some of the shared mechanisms are altered brain activity or morphology in several regions; alterations of the HPA axis; genetic susceptibilities; inflammatory signaling mediated by several cytokines, ROS, NRS, and chemokines; altered neuroglial cell functions (including indolamine dioxygenase and neurotrophic factors); dysregulation in monoamines; increase in substance P; altered galanin and opiate signaling; and excessive excitatory glutamatergic transmission and compromised GABA mediated inhibition (Maletic and Raison, 2009).

ME/CFS pathophysiological changes are strongly dependent on mitochondrial dysfunction. In particular, MAVS could protect patients with CFS from viral-related infections, which is essential in a progressive disease related to severe immune alteration (Rasa et al., 2018). The observed mitochondrial alterations are the suppression of the PDC, thereby lowering the conversion of pyruvate to acetyl-CoA and diminished mitochondrial ATP production from OXPHOS and glycolysis. In addition, the reduction in acetyl-CoA prevents melatonin-mediated cellular and mitochondrial protection. Acetyl-CoA is important for aralkylamine N-acetyltransferase (AANAT) activity, which converts serotonin to N- acetylserotonin (NAS) that is then converted to melatonin by acetyl-serotonin methyltransferase (ASMT) (Anderson and Maes, 2020). Melatonin has immune plus antioxidative effects and optimizes mitochondrial OXPHOS (Acuña-Castroviejo et al., 2017b; Anderson and Maes, 2020; McCarty et al., 2020). Other mitochondrial dysfunction mechanisms in patients suffering from ME/CFS include AMPK-altering protein recycling and fatty acid catabolism, mTORC1, and creatine kinase altering cell cycle and instability across different tissue and cell types (Khan et al., 2017; Missailidis et al., 2020). Heightened lactate production is evident in muscles, serum, and cerebrospinal fluid of ME/CFS patients following exercise, indicating significant dysregulation in exercise-induced metabolic activity, including an accelerated switching to aerobic glycolysis, increasing lactate production and suppressing ATP levels (Anderson and Maes, 2020).

Neuroinflammation is an important mechanism related to pain syndromes such as fibromyalgia and CFS. Various pathways are associated with neuroinflammation, including increased oxidative stress, peripheral inflammation, and changes in the gut microbiome. The long-lasting neuroinflammation leads to sensitization of the CNS, with chronic pain as a consequence (Chen et al., 2018; Brain et al., 2021; Chávez-Castillo et al., 2021).

Neuroinflammation in the CNS is mediated by neuroglia cells, found in high numbers in the brain and spinal cord. Glial cells (oligodendrocytes, astrocytes, microglia, and ependymal cells) can be activated by inappropriate dietary patterns, another possible cause of neuroinflammation. The most relevant diet-related risk factors for Glia-cell induced neuroinflammation are the imbalance of anti-inflammatory fatty acids (omega 3) and pro-inflammatory fatty acids (omega 6), energy-dense diets (excess sugar), a diet poor in micronutrients (vitamins and minerals), and diets low in polyphenols (Chen et al., 2018; Brain et al., 2021; Chávez-Castillo et al., 2021).

Microglia are macrophages residing in the CNS and responsible for the regulation of homeostasis. They interact dynamically with synapses and induce synaptic pruning in stages of healthy brain development. The pro-inflammatory activity microglia caused by several risk factors in modern life is an essential step in the development of neurodegenerative brain diseases, such as AD, PD, Huntington’s disease, multiple sclerosis, stroke recovery, neuropsychiatric diseases (depression and anxiety), pain, and neurodevelopmental diseases. Glia cells contribute to the pathogenesis of brain diseases through neuroinflammation. The role of mitochondrial dysfunction is central to Glial function and even development (Chen et al., 2018; Chávez-Castillo et al., 2021).

Shifts in mitochondrial metabolism are crucial in the regulation of glial immune cell phenotypes. Perhaps melatonin should be considered the main regulator of glia cell inflammatory activity. Many protective mechanisms against neuroinflammation have been attributed to melatonin functioning. Autocrine melatonin switches immune cells from a pro-inflammatory (M1) to an anti-inflammatory (M2) phenotype and produces a change in reactivity from cellular to humoral (Carrillo-vico et al., 2013; Hardeland et al., 2015; Chen et al., 2020a; Hardeland, 2021; Bitzer-Quintero et al., 2022). Besides the switch in phenotype, melatonin interacts in multiple ways with microglia. It acts as a protector and an antioxidant and, by this means, inhibits processes that cause, promote, or propagate oxidative stress and neurodegeneration, resulting in excitatory overactivity, toxicological aggressions, viral and bacterial infections, and inflammation (Carrillo-vico et al., 2013; Hardeland et al., 2015; Chen et al., 2020a; Hardeland, 2021; Bitzer-Quintero et al., 2022).

Melatonin also has several other mitochondrial mechanisms that interfere with pathways leading to chronic pain. Melatonin can eliminate mitochondrial free radicals and inhibit the activity of mitochondrial nitric oxide synthase. It restores mitochondrial calcium homeostasis, deacetylates and activates mitochondrial SIRT3, improves the integrity of the blood–brain barrier, and counteracts neuroinflammation and glutamate excitotoxicity (Morris et al., 2021). Furthermore, melatonin and its derivatives act as natural electron donors, being, therefore, highly efficient substances against oxidative stress (Manchester et al., 2015; Tan et al., 2015; Reiter et al., 2018; Salehi et al., 2019a).

The activation of the melatonin/PI3K/Akt/Bmal1-axis increases the production of neuroprotective and metabolic factors. Melatonin-induced Bmal1 drives the night-time dampening of immune cells. Under challenging conditions at night, when immune cell activity is required, an increase in pro-inflammatory cytokines suppresses pineal melatonin production, called the immune-pineal axis (Markus et al., 2021).

As already mentioned, dysbiosis is involved in many or all neurological pathologies. Dysbiosis can signal to the CNS through the production of neuromodulators, such as GABA, tryptophan, choline, serotonin, butyrate, and short-chain fatty acids (SCFA) (Rea et al., 2016; Wang and Wang, 2016; Alkasir et al., 2017; Bonaz et al., 2018; Chen et al., 2021b). Dysregulation of the gut–brain axis can contribute to the development of several neurodegenerative, neuropsychiatric, neurodevelopmental, and neuroinflammatory diseases (Mezzelani et al., 2015; Rosenfeld, 2015; Chen et al., 2016; Sampson et al., 2016; Scheperjans, 2016; Cox and Weiner, 2018; Bull-Larsen and Mohajeri, 2019). Gut microbiome-generated metabolites, such as colonic acid, D-lactic acid, D amino acids, and methyl metabolites, can pass the blood–brain barrier and modulate neuronal behavior. In this way, they influence neuronal development and brain mitochondrial dynamics. One positive pathway related to a “healthy” microbiome is via the production of SCFA that can upregulate PGC-1α as a master regulator of mitochondrial biogenesis (Petra et al., 2015; Bastings et al., 2019; Kobayashi, 2019; Theunissen et al., 2021; Taniguchi et al., 2022). Strategies to enhance melatonin production and or exogenous delivery are described in PART III.

## 4 Part III: Strategies to restore the mitochondria as a therapeutic approach for multiple chronic diseases

**FIGURE 4:**
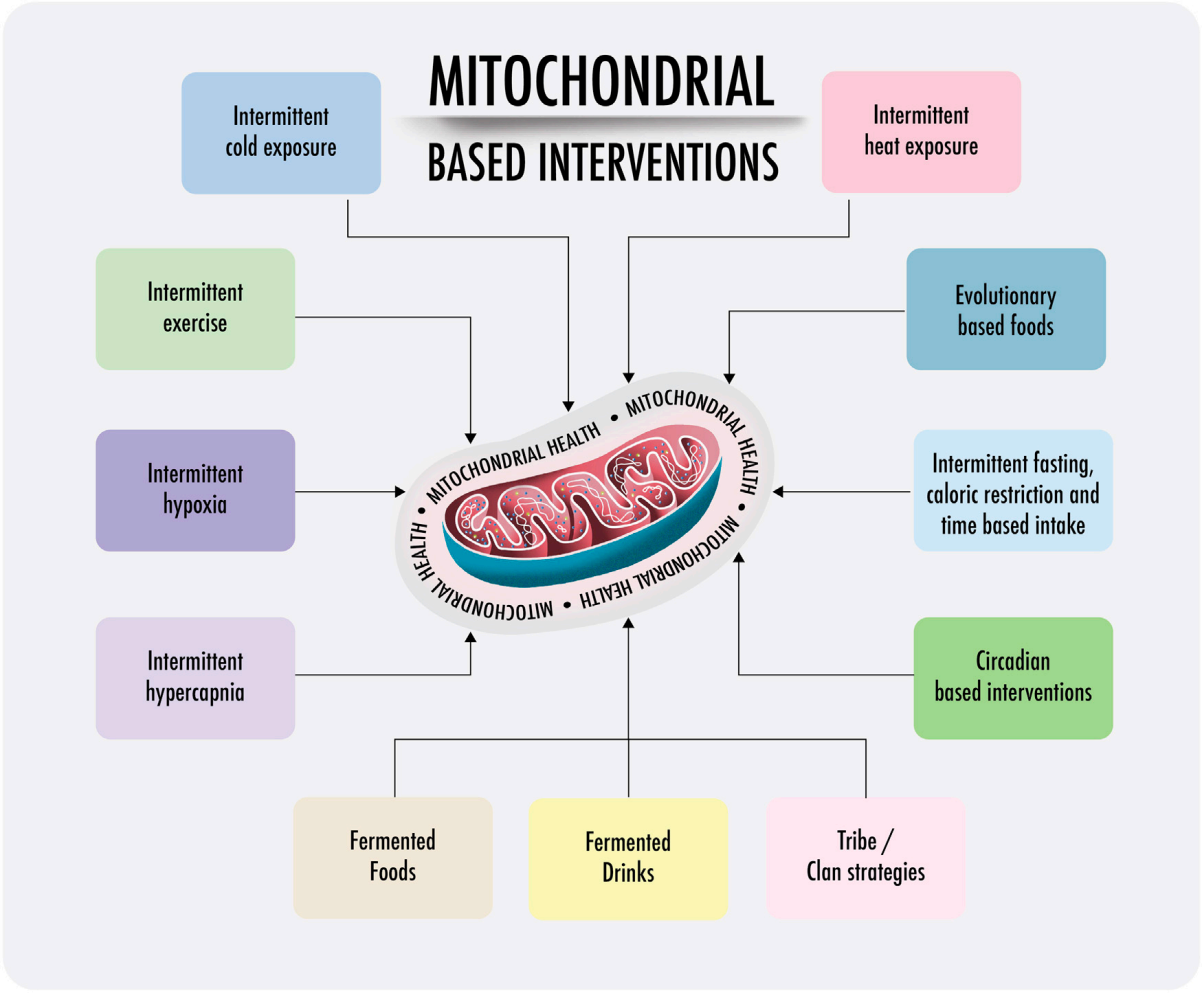
Visual representation of proposed strategies improving mitochondrial health: hormesis refers to the evolutionarily conserved adaptive responses of all living organisms to acute/temporary environmental mild physiological stress, nutritional or even voluntary challenges, through which the system modifies its tolerance to more dangerous stressors. Lifestyle interventions based on this principle are also known as hormetic strategies. At the mitochondrial level: mitohormetic strategies. Mitohormesis could support mitochondrial resilience and health, especially preventing CNCDs or premature and failed aging.

Figure 4 Understanding the different components of mitochondrial dynamics and their influence on health and disease enables the implementation of specific and targeted interventions. As mitochondria play a key role in health and disease, maintenance or recovery of mitochondrial function could or even should be considered a root cause prevention and root cause medicine.

A phenomenon called hormesis is important in the maintenance of mitochondrial health. Hormesis refers to the evolutionary conserved adaptive responses of all living organisms to mild environmental, nutritional, or even voluntary challenges, through which the system amends its tolerance to more toxic stress factors (Pruimboom and Muskiet, 2018). Subsequently, treatments and lifestyle interventions based on this principle are also known as hormetic strategies (Lee and Lee, 2019; Bongiovanni et al., 2020; Morris et al., 2021) or mitohormetic strategies at the mitochondrial level. Substances producing hormetic responses are called hormetins (Gezer, 2018; Nunn et al., 2020; Calabrese and Kozumbo, 2021). The application of hormetic strategies and combination thereof might lead to increased lifespan and disease resistance, which is evidenced in different animal studies (Schmeisser et al., 2013; Wang et al., 2019; Mattson, 2008; Berry and López-Martínez, 2020; Proshkina et al., 2020; Calabrese and Kozumbo, 2021). Hormetic triggers follow the adage “what does not kill you makes you stronger” and “if you do not use it, you lose it”; both rules might be an expectation of evolution and even a prerequisite for maintaining (mitochondrial) health.

Hormesis is the result of an upregulation of stress response genes. Triggering or stressing these mitochondrial control systems enables the expression of certain genes and results in the production of protective substances and activation of longevity-related mechanisms (Blagosklonny, 2018; Lee and Lee, 2019; Calabrese and Kozumbo, 2021).

The positive impact of the use of mitohormetic stimuli has been shown in studies on animals in early life. Mitohormetic triggers applied in early life could support mitochondrial resilience and prevent early and unsuccessful aging (Yun and Finkel, 2014; Merry and Ristow, 2016; Bárcena et al., 2018). One of the mitochondrial stress responses is characterized by the induction of specific heat shock protein (HSP) genes in the nucleus and the subsequent effects of the HSPs on mitochondrial functioning, called the mitochondrial unfolded protein response (UPRmt) (Tower, 2015; Inigo and Chandra, 2022). HSPs induce mitophagy and thus influence mitochondrial dynamics that enable the breakdown and replacement of abnormal mitochondria, including those destructed or with mutated mitochondrial genomes (Tower, 2015). The activation of HSPs and the clearance of damaged mitochondria could be a possible solution for heteroplasmy explained earlier in this document. HSPs are involved in immune function, cell cycle regulation, and proteome homeostasis. They facilitate cell function by translocating proteins to other sites within the cell, escorting proteins across cell membranes, stabilizing various proteins and receptors, and identifying and repairing damaged proteins (Brunt and Minson, 2021). HSPs are also associated with other proteins in the ER and in close proximity to the plasma membrane, nucleus, cytosol, or regulation of mitochondrial proteostasis (Brunt and Minson, 2021).

Hormetins induce responses to HSP through the stimulation of kinases and transcription factors (Lee and Lee, 2019; Nunn et al., 2020). Examples of these are the co-transcription factors PGC1α, SIRT1, and SIRT3. They code for proteins that protect mitochondrial dynamics and thus functioning. They are known to prevent the transition from acute to chronic pathologies in kidney disease models (Aparicio-Trejo et al., 2020). Restored expression of PGC-1α in cells ameliorates defective FA oxidation and corrects ATP depletion by reversing changes in damaged tissues through mitochondrial recovery (Fontecha-Barriuso et al., 2020). In addition, mitochondrial protectors and antioxidants, such as N-acetyl cysteine, are frequently used to prevent kidney damage. Their efficacy is shown in models of folic acid-induced kidney damage. They are also a good example of interventions that could support mitochondrial health and induce the production of mitochondrial antioxidants for injury prevention (Aparicio-Trejo et al., 2020; Fontecha-Barriuso et al., 2020; Zhang et al., 2021b).

Hormetic triggers also influence retrograde signaling (nuclear–mitochondrial), a phenomenon discussed earlier. Retrograde signaling influences mitochondrial dynamics, replacement, and health. Important proteins here are HIF1, nuclear factor kappa B (NFkB), PPARs, NRF1, and NRF2. All of these promote the transcription of TFAM and PGC-1α responses. They migrate and bind to target genes, eliciting the expression of cytoprotective molecules (Pruimboom and Muskiet, 2018) and facilitating mitochondrial dynamics and mitochondrial health.

Examples of hormetins as hormetic triggers are exercise, controlled oxidative stress, calory restriction (CR), temperature stressors such as heat and cold, and the use of phytochemicals. These and their relation to mitochondrial health will be discussed hereafter.

### 4.1 Nutritional strategies: Fasting, phytochemicals, phytomelatonin, and fermented food

#### 4.1.1 Fasting

Fasting is an effective way to recover mitochondrial efficiency, and multiple human studies have shown its ability to restore metabolic pathways in people suffering from chronic pathologies. Fasting regimens are promising primary and secondary prevention strategies in patients suffering from metabolic and cardiovascular diseases (Oliveira et al., 2022; Papakonstantinou et al., 2022; Vasim et al., 2022; Zang et al., 2022), pain, inflammation and immune conditions (Feng et al., 2022; Fitzgerald et al., 2022; Parveen and Alhazmi, 2022), cancer (Lv et al., 2014; Marinac et al., 2016; Taucher et al., 2022; Tiwari et al., 2022), and neurological diseases characterized by altered mood, sleep, pain, and cognition (Phillips, 2019; Brocchi et al., 2022; Caron et al., 2022; Feng et al., 2022; Lobo et al., 2022; Xu et al., 2022).

During nutrient deprivation, the fusion rate increases and the fission rate decreases, leading to increased bioenergetic efficiency (Cuevas-Sierra et al., 2019). CR, another way to create hormetic stress, induces remodeling of the ETC architecture and cristae morphology. This remodeling of the ECT architecture might be driven by ER stress (Balsa et al., 2019). Eukaryotic cells have evolved a conserved pathway called the unfolded protein response aimed to re-establish ER homeostasis. During this process, transcription factor ATF6/ATF4, protein kinase R- (PKR-) like ER kinase (PERK), and inositol-requiring enzyme (IRE1) are activated. These substances can re-establish ER homeostasis and support proper protein folding. A properly functioning ER enables the OXPHOS system to increase ATP supply and promote protein homeostasis (Balsa et al., 2019).

The combination of fasting and exercising can stimulate super complex formation through the ROS/UPR/PERK pathway. Glucose deprivation and its influence on the NADH/FADH2 gradient modulate supercomplexes, enhance mitochondrial respiration, improve all parts of the respiratory chain, and increase cristae density (Balsa et al., 2019; Cogliati et al., 2021)

Studies suggest that fasting supports the balance between the fusion and fission states and homeostasis in the mitochondrial network in multiple cells and organs (Liesa and Shirihai, 2013; Yu and Pekkurnaz, 2018). Research shows an increased expression of mitochondrial fusion-related proteins, MFN 1 and MFN 2. In an experimental model that studied the influence of CR on mitochondrial morphology and dynamics in muscle cells, it was reported that CR reduced fission (Cuevas-Cervera et al., 2022; Xu et al., 2022). CR can induce an activation of the autophagic/mitophagic machinery. As we explain above, fasting induces an ANTI-Warburg effect (Bianchi et al., 2015; Choi et al., 2016; Mattson et al., 2017; de Cabo and Mattson, 2019; Kornberg, 2020; Locati et al., 2020; Cuevas-Cervera et al., 2022) and prevents pathological accumulation or altered proteostasis that are part of the pathophysiology pathways leading to neurological and metabolic pathologies. Protein and biomass accumulation is treatable, improving autophagia, for example, with fasting (Gherardi et al., 2019; Hu et al., 2021; Lechado Terradas et al., 2021; Lizama and Chu, 2021; Roberts and Markby, 2021).

The positive effects of IF and CR and the timing of food intake on mitochondria are related to oxidative stress (Savencu et al., 2021). Numerous studies on animals and humans have shown the beneficial effects of diet interventions on mitochondria-related ROS production. Long-term exposure to CR can reduce not only oxidative stress but also the oxidative damage of the mitochondrial DNA (by 30%). Thus, CR and IF seem able to influence ROS generation and the antioxidative capacity of mitochondria (Cuevas-Cervera et al., 2022; Perez-Montilla et al., 2022).

#### 4.1.2 Phytochemicals

Nuclear erythroid-related factor 2 (NRF2) is a transcription factor encoded by the NFE2L2 gene. Currently, the activation of the NRF2 system is considered a powerful cytoprotective strategy for the treatment of different pathologies, whose pathogenesis is based on oxidative stress, including viral infections, metabolic diseases, and neurological/neuropsychiatric diseases (Bousquet et al., 2020; Bousquet et al., 2021a; Alonso-Piñeiro et al., 2021; Bhandari et al., 2021b; Wang et al., 2021b; Chun et al., 2021; Lee et al., 2021). Phytochemicals seem to reverse conditions involved in extensive lipid peroxidation, protein oxidation and carbonylation, and oxidative damage to nuclear and mitochondrial DNA (Biagiotti et al., 2019; Bhandari et al., 2021a; Morris et al., 2021; Ulasov et al., 2021; Vashi and Patel, 2021).

Optimization of the activity of NRF1 and NRF2 is a very efficient strategy for rehabilitating mitochondrial function. NRF2 activation is inversely related to mtROS generation. It increases PINK/Parkin-mediated mitophagy and removes damaged mitochondria. PGC1α, in collaboration with NRF2, increases mitochondrial biogenesis to supply healthier mitochondria. NRF2 further supports the maintenance of the mitochondrial membrane potential, OXPHOS, ATP synthesis, fatty acid synthesis, and oxidation (Buttari et al., 2022).

Under stress conditions, cells activate the regulatory enhancer sequence of the NRF2 pathway—antioxidant response element (ARE) —to promote the expression of antioxidant genes. This decreases the expression of pro-inflammatory mediators and increases the detoxifying capacity of all cell types. ARE sequences (5′-RTGACnnnGC-3′) are present in more than 200 genes, such as those that express antioxidant enzymes, NAD(P)H quinone oxidoreductase 1 (NQO1), sulfiredoxin 1 (SRXN1), glutathione S-transferase (GST), health-preserving red blood cells heme oxygenase-1 (HMOX1), multidrug resistance-associated proteins (MRP), and UDP-glucuronosyltransferase (UGT) (Buttari et al., 2022).

Phytochemicals are involved in the regulation of NRF2. They are plant antinutrients and are present in the human diet. There are thousands of different phytochemicals in commonly consumed plants. These phytochemicals can be subdivided into four classes based on their chemical structure: phenols and polyphenols, terpenoids, alkaloids, and sulfur-containing compounds (Clifford et al., 2021).

Multiple dietary phytochemicals are useful in regulating NRF and the subsequent activation of cytoprotective effects (Cuadrado et al., 2019). Some useful phytochemicals investigated are naringenin in citrus fruits and tomatoes (Lim et al., 2013). Another example is sulforaphane present in broccoli, brussels sprouts, cabbage, and cauliflower (Santín-Márquez et al., 2019; Cardozo et al., 2021; Kiser et al., 2021; Liebman and Le, 2021) and diallyl disulfide in allium plants, such as garlic and its family members (Arzanlou, 2016; Silvestris et al., 2019; Bhatwalkar et al., 2021; Jayasuriya et al., 2021).

One very powerful phytochemical is resveratrol. It modulates inflammation, improves redox balance, controls cell death and survival, mobilizes kinases, changes mitochondrial function, regulates autophagy, and affects multiple receptors, transcription factors, and ion channels, depending on the dose (Cicero and Colletti, 2015; Brown, 2017; Shetty et al., 2018; Nunn et al., 2020; Li et al., 2021). Resveratrol improves parameters in metabolic and neurodegenerative diseases by increasing antioxidative capacity via the NRF2/HO-1 pathway (Yadav et al., 2022).

Curcumin is present in turmeric root. It exhibits anti-inflammatory, antioxidant (by the action of NRF2), and anticancer activity. Various analgesic mechanisms induced by curcumin effectively attenuate pain by modulating pain-related neurotransmitters by modulating the immune response or blocking transient receptor potential vanilloid type I (TRPV1), as well as modulating purinergic receptors and chemokines, which has been shown in animal and human studies (Lim et al., 2013; Yang et al., 2014; Lv et al., 2015; Wang et al., 2016; Sato et al., 2017; Teymouri et al., 2017; Fan et al., 2018; Liu et al., 2018; Ghasemi et al., 2019; Nunn et al., 2020; Clifford et al., 2021; Jayasuriya et al., 2021; Cianciulli et al., 2022; Dasuni Wasana et al., 2022).

Quercetin is a ubiquitous plant pigment (flavonoid). It is found in many plants and foods, such as onions, green tea, apples, berries, *Ginkgo biloba*, St. John’s wort, elderberry, and buckwheat tea. Meta-analyses show significant positive results with regard to physical resistance, lower mortality, fewer complications from respiratory viral infections, reduction in pro-inflammatory cytokines and chemokines, a decrease in the amount of ROS, and optimization of mucus production. Quercetin efficiently reduces triglycerides at doses greater than 50 mg/day. It has widely studied neuroprotective effects against neurotoxic chemicals and can also prevent the progression and development of neuronal injury and neurodegeneration (Kressler et al., 2011; Serban et al., 2016; Shi and Williamson, 2016; Sahebkar, 2017; Ho et al., 2018; Bousquet et al., 2020; Nunn et al., 2020; Zhang et al., 2020; Alasmari, 2021; Bhat, 2021; Brito et al., 2021; Ghafouri-Fard et al., 2021; Pinheiro et al., 2021; Soofiyani et al., 2021; Yi et al., 2021).

Ginsenosides in ginger roots in AD models activate NRF2 and suppress ROS/ASK-dependent mitochondrial apoptosis (Liu et al., 2019). Ginsenoside Rd inhibits proliferation and reverses resistance to cisplatin in cancer models (Chian et al., 2019). Pinostrobin protects against neurotoxins by activating NRF2/ARE in Parkinson’s models and ellagic acid in AD models. It has a neuroprotective effect through the NF-κb/NRF2/TLR4 pathway (Grilc et al., 2021).

The use of NAC, coenzyme Q10, resveratrol, and melatonin also increases the activity of NRF2 (Oppedisano et al., 2020; Morris et al., 2021). For improving brain health in neurological disorders, the intake of NAC is proposed and is associated with better brain mitochondrial function and decreased oxidative stress, reduced neuroinflammation, relief of ER stress, and regulation of the unfolded protein response (Morris et al., 2021). Q10 acts as a superoxide scavenger in neuroglial mitochondria, instigates mitohormesis, enhances lipid peroxidation in the IMM, activates uncoupling proteins, promotes mitochondrial biogenesis, is important to restore mitochondrial damage after statins consumption, and has positive effects on the plasma membrane redox system (Lee et al., 2013; Banach et al., 2015; Mohammadi-Bardbori et al., 2015; Morris et al., 2021; Pradhan et al., 2021; Rauchová, 2021).

#### 4.1.3 Melatonin: Phytomelatonin

Melatonin can eliminate mitochondrial free radicals, inhibit mitochondrial nitric oxide synthase, restore mitochondrial calcium homeostasis, and further deacetylate and activate mitochondrial SIRT3 (Galano et al., 2018; Morris et al., 2021). Melatonin improves PGC-1α, Mfn2, and mitochondrial parameters in an animal model of fibromyalgia (Favero et al., 2019). Melatonin enhances the integrity of the blood–brain barrier and the integrity of the intestines and counteracts neuroinflammation and glutamate excitotoxicity (Morris et al., 2021).

We described the functions of melatonin as a mitochondrial protector in an earlier section. Blood melatonin concentrations increase significantly after the consumption of food containing melatonin (Salehi et al., 2019a). Melatonin can be found as a bioavailable substance in many foods. Animal products such as fish and eggs are relatively rich sources of melatonin (Meng et al., 2017a). In plant foods, nuts are an interesting source of melatonin. Mushrooms, berries, cherries, tomatoes, and cucumbers, among many other fruits and vegetables, also contain melatonin (Meng et al., 2017b; Salehi et al., 2019a). Studies on 108 herb species have revealed the presence of melatonin in concentrations ranging from a few to several thousand nanograms per gram of tissue, meaning they are potentially good natural sources of this molecule (Salehi et al., 2019b). Melatonin has also been detected and quantified in roots, shoots, leaves, flowers, fruits, and seeds, but its highest levels have been found in seeds (Salehi et al., 2019b). In the scientific literature, the term phytomelatonin sometimes appears, mainly applied to refer to endogenous melatonin synthesized by plants. Interestingly, in medicinal plants, including pyrethrum maruna (*Tanacetum parthenium* L.) and *Hypericum perforatum* L., melatonin levels are sometimes higher than those found in animals (Salehi et al., 2019b)

#### 4.1.4 Fermented food items

As described earlier, intestinal permeability and dysbiosis are risk factors for mitochondrial dysfunction. One simple strategy to improve our gut microbial diversity and intestine integrity is the consumption of fermented food items (Tamang et al., 2016; Dimidi et al., 2019; Melini et al., 2019; Stiemsma et al., 2020). The ability of fermented foods to prevent disease may be related to the number of beneficial microbes, high content of sulforaphane (in, e.g., cabbage and its family), and capsaicinoids (a spicy substance in red pepper powder) (Kwak et al., 2014; Patra et al., 2016; Kim et al., 2018; Woo et al., 2018; Park et al., 2019; Han et al., 2020; Cantadori et al., 2022). The fermentation process increases the number of phenolic compounds (Le et al., 2020) and is a good source of micronutrients, GABA, and amino acids (Lin, 2013; Tamang et al., 2016; Lee et al., 2018; Bousquet et al., 2021b). Many active substances in fermented food items possess potent antibacterial, anticancer, and immune-enhancing capacities (Lin, 2013; Tamang et al., 2016; Lee et al., 2018; Bousquet et al., 2021b). Fermented vegetables and spices are NRF2-induced enzyme agonists. The synergy between fermented food items and spices shows activation effects on NRF2 and a potent antioxidative effect. At the same time, spicy foods are likely to desensitize TRP channels and act in synergy with exogenous polyphenols and flavonoids in plants that activate the NRF2 pathway (McKenzie et al., 2017; Kim et al., 2018; Lee et al., 2018; Woo et al., 2018; Dimidi et al., 2019; Park et al., 2019; Bousquet et al., 2020). Undoubtedly, the most important feature of kimchi, a fermented food item, is its extraordinary effect with regard to the improvement of the gut microbiome, improving the absorption and production of substances essential for health by the bacteria in the gastrointestinal system (Dimidi et al., 2019; Han et al., 2020; Stiemsma et al., 2020).

### 4.2 Exercise

Contraction of skeletal muscle during exercise activates many hormetic pathways, including improved nutrient sensor AMP kinase activity, mTOR regulation, intracellular PI3K-Akt activity, transcriptional PGC-1α, and sirtuin 1 signaling. All these mechanisms orchestrate the adaptions involved in response to exercise (Foster et al., 2015; Merry and Ristow, 2016; Roberts and Markby, 2021; Magaña et al., 2022). Exercise improves macro-autophagic and lysosomal activity and protects the balance between mitochondrial biogenesis, mitochondrial dynamics, and mitophagy (Sorriento et al., 2021). Mitochondrial response to exercise follows multiple mechanisms (Tian et al., 2022): increase in mitochondrial biogenesis (Wallace, 2015); improvement of expression and action of proteins involved in the mitochondrial dynamics through mitofusins (fusion and fission proteins, promoting a more fused, tumular network) such as OPA1 (Navarro-Ledesma et al., 2022); increase in mitochondrial turnover by the action of proteins related to autophagy and mitophagy (e.g., PINK1, parkin, nix, and Bnip3) and (Pruimboom et al., 2016) quality control supporting mechanisms through the degradation of damaged or dysfunction mitochondria; and mitochondrial cristae remodeling or shaping (Ruiz-Núñez et al., 2013), improving chain complexes and mitochondrial respiratory efficiency (Roberts and Markby, 2021; Sorriento et al., 2021; Lv et al., 2022). Exercise impacts the stoichiometry of the SCs, enhancing the efficiency of electron flux by segmentation of the CoQ pool, improving the stability of the individual respiratory complexes, and avoiding ROS excess (by CI and CIII) (Huertas et al., 2021; Roberts and Markby, 2021). Theoretically, exercise per its capacity to create ROS as a signaling molecule could also impact signaling pathways linked to quality and quantity control of mitochondria (Barbieri et al., 2015; Axelrod et al., 2019). Training volume strongly correlates positively with changes in mitochondrial volume. Therefore, a mix between high-intensity interval training (HIIT) and endurance training seems important (Bishop et al., 2014; Barbieri et al., 2015; Stawski et al., 2017).

### 4.3 Cold exposure

Cold exposure promotes increased expression of mitochondrial biogenesis. The use of short-lasting cold triggers has been shown to increase the number and activity of mitochondria and protection against oxidate damage (Saltykova, 2019). The influence of cold exposure on mitochondria has been mentioned in relation to the so-called browning of adipose tissue: the transition of the white adipose tissue into a beige or brown adipose tissue (BAT) (Saito et al., 2009). When white adipocytes are re-programmed to beige adipocytes, they show an increase in mitochondria. PGC-1α is considered the link in the transition from external physiological stimuli (e.g., cold) to an internal metabolic response, such as mitochondrial biogenesis (Ihsan et al., 2021). Cold exposure activates UCP1-mediated thermogenesis in adipose tissue, and UCP-1 is mainly found in adipocytes of BAT. Uncoupling proteins play a vital role in regulating mitochondrial membrane potential, preventing ROS production, and regulating calcium homeostasis, amongst others (Saito et al., 2009; Chung et al., 2017; Okamatsu-Ogura et al., 2020).

### 4.4 Heat exposure

Heat is typically used to improve heat tolerance, although it has also been shown to improve physical performance (Waldron et al., 2021).

Heat exposure is often investigated using a traditional wood, electric, or infrared sauna. Some heaters heat the air by raising the temperature. Traditional saunas use temperatures of 70°C–100°C. Infrared heaters emit thermal radiation, which heats the body directly using 45°C–60°C. Dry sauna humidity is 10%–20%, whereas the humidity levels in steam saunas are above 50% (Patrick and Johnson, 2021).

Exposure to high temperatures is a hormetic stress stimulus, a robust response that mainly affects the skin and the cardiovascular system. Cardiac output can increase by as much as 60%–70%. During sauna exposure, 50%–70% of the body’s circulation is redistributed to the skin to facilitate sweating, resulting in fluid losses at a rate of approximately 0.6–1.0 kg/h through sweating to cool the body and avoid rapid increases in its core temperature. It also detoxifies and eliminates heavy metals (Patrick and Johnson, 2021).

The molecular response to heat includes increased expression of HSPs, transcriptional regulators, such as NRF2, and pro- and anti-inflammatory factors (Patrick and Johnson, 2021).

In cancer models, at temperatures of 40°C, one sees an increase in the expression of the transcription factor protein NRF2. The same holds for SOD, catalase, heme oxygenase-1, glutamate cysteine ligase, and HSP70 (Glory and Averill-Bates, 2016). Increased NRF2 expression and catalase activity at 40°C were inhibited by the antioxidant PEG-catalase and the p53 inhibitor pifithrin-α, suggesting that mild thermotolerance (40°C) increases prooxidant levels, which in turn activates NRF2 (Glory and Averill-Bates, 2016).

Chronic high temperatures can cause the inactivation of protein synthesis and DNA repair processes. In that situation, cells die by apoptosis and/or necrosis or become sensitized to other cytotoxic modalities, such as radiation. Intermittent hyperthermia (41°C–45°C) activates apoptosis through the mitochondrial death receptor and ER pathways. Training cells at elevated temperatures induces thermotolerance (Glory et al., 2014). Both exercise and sauna use raise core body temperature and acutely increase plasma IL-6 and IL-10 levels. IL-6 exerts anti-inflammatory properties through the activation of IL-10 (Patrick and Johnson, 2021).

### 4.5 Breathing techniques

Many other strategies based on evolutionary challenges have been investigated. In particular, the strategies that involve gas changes, respiratory changes, or respiratory techniques, such as pranayama, are promising as interventions for primary and perhaps secondary prevention for the development of chronic diseases.

Studies on participants engaging in pranayama breathing have shown psychological and physiological benefits in cancer, cardiovascular diseases, and mainly pulmonary diseases (Jayawardena et al., 2020). Pranayama breathing also shows positive effects with regard to the improvement of exercise tolerance in patients with pulmonary diseases (Kaminsky et al., 2017). Similar effects are also shown in patients with asthma, improving their quality of life (Erdoğan Yüce and Taşcı, 2020; Das et al., 2022). Yoga respiratory practice significantly reduces the levels of anxiety and negative affect. Its effects are thought to be caused by the modulation of activity and connectivity in brain areas involved in attention, awareness, and emotion processing measured with functional MRI (Novaes et al., 2020). Slow types of yoga breathing techniques show beneficial effects on cardiovascular and autonomic variables, unlike fast breathing (Nivethitha et al., 2016). Different techniques will produce different effects, although the mechanisms are not yet fully understood (Nivethitha et al., 2016). Moreover, intermittent hypobaric hypoxia (IHH) and endurance training (ET) are protective strategies for improving stress resistance. Again, mitochondrial modulation is an important step in this process (Magalhães et al., 2013).

After hypoxia exposure, animal models show mitochondrial-resistant phenotypes and a protective hypoxia response against mitochondrial toxicity (Jain et al., 2016). Intermittent hypoxia training (IHT) in animal models significantly improved OXPHOS and α-ketoglutarate oxidation (Kurhaluk et al., 2013). Moreover, IHT reorganizes the mitochondrial energy metabolism in the liver (Kurhaluk et al., 2013). IHT induces an adaptative specific type of mitochondrial mitosis in cardiac and lung tissue depending on the duration (Rozova and Mankovska, 2012). IHT is often investigated in 20-day programs of 5–8 daily cycles of 5–10 min moderate intense hypoxia (9.5%–10% O_2_) and 4 min exposures to normoxia (21% O_2)_, with each daily session totaling 45–98 min (Jung and Mallet, 2018). Proven cardiovascular function improvements are shown, as well as increases in resistance against toxins. It is further proposed as a powerful, non-invasive brain protective strategy (Jung and Mallet, 2018). Acute intermittent hypoxia (AIH) is one of the most promising approaches to improve recovery after spinal cord injury and other diseases with motoneurons alterations due to its effects on neuronal plasticity through increasing VEGF, brain-derived neurotrophic factor (BDNF), and phosphorylated and non-phosphorylated forms of the BDNF receptor tropomyosin-related kinase B (TrkB) (Hassan et al., 2018; Vose et al., 2022). AIH combined with task-specific training synergistically improves motor function (Welch et al., 2020). AIH improves cognitive and mitochondrial functions and protects against cerebrovascular malfunction in neurological diseases (Manukhina et al., 2016; Serebrovska et al., 2019; Burtscher et al., 2021).

## 5 Mitochondrial health, mitochondrial dynamics, mitophagy, and biogenesis

### 5.1 Mitochondrial health

The opposite of disease is health. Understanding what mitochondrial health means and how to maintain mitochondrial health enables the consistent execution of a myriad of functions and thus contributes to prevention, care, and cure in times of pathologies.

Mitochondria are highly dynamic organelles that undergo frequent structural and metabolic changes to fulfill cellular demands. Mitochondrial dynamics depend mainly on mitochondrial fission, fusion, biogenesis, and mitophagy. These processes coordinate the control of mitochondrial morphology, quantity, quality, turnover, and inheritance (Yapa et al., 2021; Green et al., 2022). Maintenance of both mitochondrial quantity and quality is strictly related to the conservation of an adequate concentration of several proteins, such as PGC-1α, TFAM, mitochondrial cristae biogenesis protein optic atrophy 1 (Opa1), Drp1, Mitofusin 1 (Mfn1), Mitofusin 2 (Mfn2), mitochondrial fission protein 1 (FIS1), PINK1, Parkin, VDAC1, Bnip3, and Nix, reunited in the process of proteostasis (Moreira et al., 2017; Wiese and Bannister, 2020; Lima et al., 2022).

### 5.2 Mitochondrial dynamics: Fusion and fission

A fine-tuned balance needs to exist between fusion and fission (Green et al., 2022). Fusion means the physical merging of mitochondrial segments of two originally distinct mitochondria. Amongst others, it takes care of the replenishment of damaged mitochondrial DNA. The main proteins responsible are Mfn1, Mfn2, and OPA1 in IMM and lipid components (e.g., cardiolipin) (Adebayo et al., 2021).

Conversely, fission is a division of mitochondria performed in the ER. It is required for mitochondrial motility and mitochondrial inheritance in the G2/M phase of the cell cycle. It influences the regulation of mitochondrial size and shape and the distribution of mitochondria throughout the cell body. The main proteins involved in fission are dynamin-related protein 1 (DRP1), dynamin-1 (DNM1), DNM2, and FIS1 (Quiles and Gustafsson, 2022). Some studies have found that upon aging, mitochondria tend to be more fragmented, suggesting that fusion is decreased and/or fission is increased (Wang et al., 2019). Fused mitochondria have indeed been suggested as being metabolically more active than fragmented mitochondria, as cells with a fused mitochondrial network seem to have a higher respiratory rate than cells with fragmented mitochondria (Westermann, 2012).

### 5.3 Mitophagy and biogenesis

Mitochondrial biogenesis, a cellular process enabling the production of new mitochondria, is mediated by different activators, regulators, and transcription factors, such as PGC-1a and NRF2. Damaged mitochondria are removed via a selective autophagosomal process called mitophagy. The major regulator for this is PGC1 (Ma et al., 2020; Choubey et al., 2021; Terešak et al., 2022). Aging has been associated with decreased mitophagy capacity and mitochondrial biogenesis, leading to the accumulation of mitochondrial damage (Chen et al., 2020b).

Although mitochondria are a source of ROS, they are vulnerable to oxidative stress. Therefore, endogenous anti-oxidative systems play an important role in cell survival under physiological and pathological conditions. NRF2 is important in this defense. The NRF2/ARE signaling pathway affects almost all mitochondrial processes and is, therefore, an important target in the protocols proposed to improve mitochondrial health (Khan et al., 2021b; Chen, 2021; Gilardini Montani et al., 2021; Herengt et al., 2021; Liebman and Le, 2021; Matsumaru and Motohashi, 2021; Syed et al., 2021; Thiruvengadam et al., 2021; Ulasov et al., 2021; Zgorzynska et al., 2021; Zhao et al., 2021). In order to cope with deleterious effects, mitochondria feature different mechanisms for quality control. One such mechanism is the mitochondrial unfolded protein response (UPR^mt^), which corresponds to the transcriptional activation of mitochondrial chaperones, proteases, and antioxidant enzymes to repair defective mitochondria (Inigo and Chandra, 2022).

Interventions explained above could serve the purpose of mitochondria anatomical and functional maintenance. This review describes a series of possible treatment options as primary and secondary preventive interventions in mitochondria dysfunction-related disorders and diseases.

## 6 Conclusion

Modern life has come with novel risk factors. Although our adapted brain produced the modern and novel environment, our immune system and metabolism have not yet adapted to those new circumstances. Many novel risk factors, such as sitting time, lack of physical activity, food abundance, sleep disturbances, and lack of micronutrients, and environmental factors, such as pollution, can produce severe mitochondrial stress, damage, and dysfunction. The metabolic disturbances caused by mitochondrial dysfunction and damage caused by the aforementioned risk factors are hallmarks of most, if not all, of the metabolic, immune, neurological, and infectious diseases and CNCDs in general. Risk factors and consequences can be prevented and/or treated, considering that one of the main pathways is the switch from OXPHOS to glycolysis directed by mTOR. The chronic Warburg effect leads to proliferative, inflammatory, or fibrotic states, increasing the intracellular abundance of biomass. In this study, we describe the immuno-metabolic mechanism that impedes healthy OXPHOS. Evolutionary medicine provides us with mitohormetic strategies. These are simple, low-risk, and economically cheap strategies based on enhancing human molecular responses through epigenetic mechanisms. Intermittently applied and in the right combination, they provide powerful tools to facilitate mitochondrial health through several pathways. As mitochondrial functioning is essential in understanding health and disease, the use of hormetic interventions, protecting or recovering normal mitochondrial functioning, could result in widespread health benefits and possibly be effectual in primary and secondary chronic disease prevention. These so-called hormetic strategies support immune system efficiency and an anti-inflammatory phenotype, along with metabolic and neurological plasticity. Avoiding metabolic glycolytic states and switching to OXPHOS is especially described in fasting (an anti—Warburg strategy). Other promising hormetic strategies are exposure to intermittent therapeutic cold or heat. Nutrition as medicine with regard to mitochondrial health is evidenced by numerous studies related to the beneficial effects of phytochemical-rich food and fermented food items. Breathing techniques, including therapeutic intermittent hypercapnia and hypoxia, can even serve as quick wins in people suffering from pain and fatigue. All these robust strategies trigger evolutionarily conserved mechanisms, which, in turn, make *homo sapiens* more resilient and thus resistant to the toxic effects of modern life. Mechanisms behind each hormetic trigger strategy are not fully understood, and further research is needed, not only *in vitro* and *in vivo*, but also in clinical settings with humans.

## Figures and Tables

**Table:** 

**Acetyl-CoA**	Acetyl coenzyme A
**ADP**	Adenosine diphosphate
**AGE**	Advanced glycation end-products
**AHSG**	Alpha-2-Heremans-Schmid Glycoprotein
**AKI**	Acute kidney injury
**AMPK**	AMP-activated protein kinase/adenosine monophosphate protein kinase
**ARNTL**	Aryl hydrocarbon receptor-like nuclear translocator
**ATCase**	Aspartate transcarboxylase
**ATP**	Adenosine triphosphate
**bHLH-PAS**	Basic helix–loop–helix/Per-ARNT-SIM
**BMAL1**	Basic helix–loop–helix ARNT-like 1
**Ca** ^**2+**^	Calcium ions
**CAD**	Dihydroorotase
**CK1ε**	Casein kinase I isoform epsilon
**CKD**	Chronic kidney disease
**Clock**	Circadian locomotor output cycles kaput
**CLR**	C-type lectin receptors
**CNCD**	Chronic non-communicable diseases
**CNS**	Central nervous system
**COX**	Cytochrome C oxidase
**CPS2**	Carbamoyl-phosphate synthetase 2
**CRY**	Cryptochrome
**Cry 1**	Cryptochrome circadian regulator 1
**Cry 2**	Cryptochrome circadian regulator 2
**CVD**	Cardiovascular diseases
**DAMP**	Damage-associated molecular pattern
**DKD**	Diabetic kidney disease
**DNA**	Deoxyribonucleic acid
**ECT**	Electron transport chain
**EDCs**	Endocrine disrupting chemicals
**eGFR**	estimated glomerular filtration rate
**ER**	Endoplasmic reticulum
**ETF**	Electron-transferring flavoprotein
**FA**	Fatty acid
**FAD**	Flavin adenine dinucleotide
**FAO**	Fatty acid oxidation
**Fe-S**	Iron + sulfur
**Fe2+**	Ferrous ion
**FIS1**	Fission protein 1
**FTH**	Ferritin heavy chain
**FTL**	Ferritin light chain
**FtMt**	Mitochondrial ferritin
**GABA**	Gamma-aminobutyric acid
**GAPDH**	Glyceraldehyde-3-phosphate dehydrogenase
**GLUT**	Glucose transporter
**GSH**	Glutathione
**GSSG**	Glutathione disulfide
**GTP**	Guanosine triphosphate hydrolases
**H**_**2**_ **O**_**2**_	Hydrogen peroxide
**HD**	Huntington disease
**HIF1**	Hypoxia-inducible factor 1
**hsCRP**	High-sensitivity C-reactive protein
**HSP**	Heat shock protein
**IFN**	Interferon
**IL**	Interleukin
**IL**	Intermittent living
**IMM**	Inner mitochondria membrane
**JAK/STAT**	Janus kinase/signal transducer and activator of transcription
**LDH**	Lactate dehydrogenase
**LDL**	Low-density lipoprotein
**LGI**	Low-grade inflammation
**LOF**	Loss of function
**LPS**	Lipopolysaccharide
**m-L-LDL**	Mitochondrial lactate dehydrogenase
**MAM**	Mitochondria-associated membrane
**MAVS**	Mitochondrial antiviral-signaling protein
**MCP**	Monocyte chemoattractant protein
**MCT**	Monocarboxylate transporters
**MCU**	Mitochondrial Ca2+ uniporter
**MDP**	Mitochondrial derived proteins
**MetS**	Metabolic syndrome
**MFF**	Mitochondrial fission factor
**Mfn**	Mitofusin
**miRNAs**	microRNS
**MMPT**	mitochondrial membrane permeabilization transition
**MOM**	Mitochondrial outer membrane
**MOTS**	Mitochondrial 12S ribosomal RNA type c open reading frame
**mPTP**	Mitochondrial permeability transition pores
**mRNA**	Messenger RNA
**MSR**	Mitochondrial stress response
**MT1**	Melatonin 1
**mtDNA**	Mitochondrial DNA
**MTHFD2**	Methylenetetrahydrofolate dehydrogenase 2
**mTOR**	Mechanistic target of rapamycin
**NA**	Noradrenaline
**NAC**	N-acetyl-cysteine
**NAD**	Nicotinamide adenine dinucleotide
**NAD+**	Nicotinamide adenine dinucleotide
**NADL**	Non-alcoholic fatty liver disease
**NADPH**	Nicotinamide adenine dinucleotide phosphate
**NcRNAs**	Non-coding RNAs
**NF-κB**	Nuclear factor kappa B
**NGF**	Nerve growth factor
**NLR**	NOD-like receptors
**NLRP3**	NLR family pyrin domain containing 3
**NNT**	Nicotinamide nucleotide transhydrogenase
**NPAS2**	Neuronal PAS domain protein 2
**NPY**	Neuropeptide Y
**NR1D**	Nuclear receptor subfamily 1 group D
**NR1D1**	Nuclear receptor subfamily 1 group D1
**NR1D2**	Nuclear receptor subfamily 1 group D2
**NRF**	Nuclear respiratory factor
**OCR**	Oxygen consumption rates
**OM**	Outer mitochondria membrane
**ONOO**	Peroxynitrite
**OPA1**	Protein optic atrophy 1
**OXPHOS**	Oxidative phosphorylation
**PAMPS**	Pathogen-associated molecular patterns
**PARP**	Poly ADP ribose polymerase
**PD**	Parkinson disease
**PDC**	pyruvate dehydrogenase complex
**PDc**	Pyruvate dehydrogenase complex
**Per 1**	Peroxisome 1
**Per 2**	Peroxisome 2
**Per 3**	Peroxisome 3
**PGC-1α**	Peroxisome proliferator-activated receptor gamma coactivator-1 alpha
**Pi**	Inorganic phosphate
**PI3K**	Phosphatidylinositol 3-kinase-protein kinase B
**PINK**	PTEN-induced putative kinase 1
**PKC-β2**	Protein kinase C beta 2
**PPARγ**	Peroxisome proliferator-activated receptor-γ
**PRR**	Pattern-recognition receptors
**RAR**	Related orphan receptor A
**RBP**	Retinol binding protein
**Rev-erbα**	Rev-Erb alpha
**RLR**	(RIG-1)-like receptor
**RNA**	Ribonucleic acid
**ROR**	Receptor tyrosine kinase-like orphan receptor 1
**ROS**	Reactive oxygen species
**SCN**	Suprachiasmatic nucleus
**SGLT2**	Sodium-glucose cotransporter-2
**SIRT**	Sirtuin
**SNP**	Single nucleotide polymorphisms
**SOCS1/3**	Cytokine signaling 1 and 3
**SOD**	Superoxide dismutase
**sORF**	short open-reading frames
**STING**	Stimulator of interferon genes
**T1D**	Type 1 diabetes
**TCA cycle**	Tricarboxylic acid cycle
**TFAM**	Transcription factor A, mitochondrial
**TFB2M**	Transcription factor B2, mitochondrial
**TGF**	Transforming growth factor
**TLRs**	Toll-like receptors
**TNF**	Tumor necrosis factor
**Tug1**	Taurine upregulated gene 1
**UCP2**	Mitochondrial uncoupling protein 2
**UDP-GlcNAc**	UDP-N-acetyl-glucosamine
**UPR**	Unfolded protein response
**VEGF**	Vascular endothelial growth factor
**wt-mtDNA**	Wild-type mitochondrial DNAANN.
